# The impact of evolutionary dynamics and expression plasmid stability on long-term heterologous protein production by yeast

**DOI:** 10.1186/s12934-026-03071-1

**Published:** 2026-07-23

**Authors:** Mingzhi Xu, Andrei Parker, Klaus Winzer, Simon V. Avery, Christopher Finnis

**Affiliations:** 1 School of Life Sciences, University of Nottingham, University Park, Nottingham, NG7 2RD UK; 2 Phenotypeca Limited, BioCity Nottingham, Nottingham, NG1 1GF UK; 3 School of Life Sciences, Biodiscovery Institute, University of Nottingham, University Park, Nottingham, NG7 2RD UK

## Abstract

**Background:**

Baker’s yeast, *Saccharomyces cerevisiae*, is a widely used industrial host organism for heterologous protein production. Ensuring stable, high-level production over extended production campaigns is important for low-cost manufacturing. However, maintaining consistent yields over multiple generations is challenging as strain productivity may decline over time. Understanding potential evolutionary mechanisms underlying such decline is therefore important for optimising yeast-based expression systems in continuous manufacturing.

**Results:**

In this study, diverse yeast libraries carrying high-copy-number whole-2-micron episomal expression plasmids producing a secreted SARS-CoV-2 spike protein fragment fused to mCherry were used to investigate fluorescence-based production stability during prolonged cultivation in yeast. We observed that while some strains maintained relatively stable mCherry fluorescence, most strains with high initial total fluorescence showed marked declines over 140 generations. Growth assays indicated that cultures with reduced mCherry fluorescence had a fitness advantage over high-fluorescence cultures. The decline was partly explained by phenotypic heterogeneity among genetically uniform cells; early-stage (10th generation) cultures contained distinct, non-fluorescent, low-fluorescence and high-fluorescence subpopulations (these subpopulation phenotypes were non-heritable), whereas evolved low-fluorescence cultures after 140 generations displayed loss of the high- and/or low-fluorescence subpopulations and reduced cell-to-cell heterogeneity. Despite this, the selected evolved low-fluorescence isolates had fluorescence restored to near-initial levels after being cured of their expression plasmids and retransformed with plasmids from early-stage, high-fluorescence cultures, indicating that, in the selected evolved low-fluorescence isolates analysed, plasmid-associated changes were sufficient to explain the stable reduction in mCherry fluorescence. Whole-plasmid sequencing of the episomal 2-micron expression plasmids isolated from low-fluorescence cultures at the 140th generation revealed mutations in key functional regions, including the methionine-regulated *MET17* promoter driving SARS-CoV-2 protein expression, *FRT* recombination sites and the SARS-CoV-2 spike protein fragment coding sequence.

**Conclusion:**

The results support a model in which prolonged expression of a burdensome secreted recombinant protein can be associated with early non-genetic heterogeneity in intracellular mCherry fluorescence, growth advantages of low-fluorescence cells and evolution of plasmid variants that reduce expression. The extent of fluorescence decline varied among genetically diverse strains, with some remaining comparatively stable, highlighting the potential of exploiting yeast strain diversity to obtain production strains with improved long-term stability.

**Supplementary Information:**

The online version contains supplementary material available at https://doi.org/10.1186/s12934-026-03071-1.

## Background

Baker’s yeast, *Saccharomyces cerevisiae*, has been widely used in industrial manufacturing owing to its strong track record of safety for FDA-approved biologics (i.e. therapeutic biological medicines such as recombinant proteins and vaccines), rapid growth, high production yields and ease of manipulation [1, 2]. Advances in recombinant DNA technologies, genetic engineering and synthetic biology have further enhanced the value of yeast-based systems for biologics production [2]. Nowadays, *S. cerevisiae* is one of the most widely used expression systems for the production of high-value products ranging from pharmaceuticals, including hepatitis and HPV vaccines, insulins and semaglutide precursor, to fragrances such as vanillin [3–7]. The global biopharmaceutical market size is over 500 billion USD, with more than 200 billion USD attributed to recombinant proteins, especially antibodies [8]. This market continues to expand rapidly at an annual growth rate of 8.5%, with about 15% of all biopharmaceutical proteins for human use being produced using *S. cerevisiae* as an expression chassis [7–9]. However, maintaining stable, high-level production over prolonged campaigns, particularly in continuous processes, remains a key biomanufacturing challenge. Given the benefits of continuous culture for cost reduction during biopharmaceutical manufacturing, selecting highly stable production strains is desirable.

Previous studies have indicated several potential causes of production instability in yeast-based systems for bioproduction, including metabolic burden, plasmid instability, mutational changes and stress responses [10–15]. High-productivity strains appear particularly susceptible to these challenges, and product overexpression can be linked to a higher metabolic cost and burden in the cells [13]. Consequently, high-fluorescence cell populations in this study, where mCherry fluorescence is used as a proxy for recombinant SARS-CoV-2-spike-fragment-mCherry protein production, may become heterogeneous over time, leading to mixed phenotypes with varying production levels [16–18]. The metabolic burden of high-level production can negatively impact growth rates, thereby conferring a selective advantage to low-fluorescence or non-fluorescent subpopulations [19]. Such population dynamics would ultimately result in a decline in overall productivity after sufficient generations. However, while these potential contributing factors have been reported, few studies have systematically tracked the molecular and evolutionary changes occurring during long-term bioproduction. Some relevant studies have focused on laboratory strains, which are not ideal models for simulating the diversity and evolutionary development that may arise in real industrial settings [11, 12, 20–22]. Gaining insight into how yeast populations evolve at the molecular level during prolonged fermentation is crucial for developing robust, high-performing production systems. Here, a SARS-CoV-2 spike fragment-mCherry protein was selected as a case study of a protein with a high metabolic burden to identify the worst-case productivity declines to enable analysis of the underlying processes.

Recent efforts have focused on engineering more efficient and resilient yeast cell factories for industrial use [23–25]. However, the yeast strains currently used in industry are often neither the best-performing nor the most suitable, with many used for historical reasons rather than through careful selection and design for a specific application [26, 27]. Advances in yeast population genomics have revealed that *S. cerevisiae* harbours enormous natural genetic diversity, which offers a reservoir of traits for selection [27]. Combining this natural strain diversity with rational strain development provides an opportunity to select genotypes better suited for industrial applications [26, 28].

To investigate how high-level recombinant protein production impacts population dynamics and genetic stability, we established an experimental system in which production cultures were repeatedly passaged into fresh medium upon reaching stationary phase, thereby simulating key aspects of an extended production campaign, while recognising significant differences between large-scale industrial continuous fermentation. Specifically, we aimed to: (1) determine how the initial production level relates to long-term production stability and diversification; (2) identify dominant factors, genotypic or non-genetic (i.e. phenotypic heterogeneity among genetically uniform cells), associated with expression declines during prolonged cultivation; (3) characterise the spectrum and functional consequences of mutations arising during prolonged cultivation. Our findings provide insight into the relationship between production burden and population evolution, offering an avenue to improve the robustness of yeast-based expression systems for larger-scale industrial applications, particularly by selecting optimal production strains from genetically diverse libraries.

## Materials and methods

### Strains, culture conditions and DNA transformation

Strains were derived from two libraries provided by Phenotypeca Ltd. First, a haploid library with the core genotype of *hoΔ::HphMX; leu2Δ; ura3Δ; pep4Δ::KanMX* for all segregants. Disruption of the *HO* locus with *HphMX* was used to prevent mating-type switching and self-mating, thereby maintaining stable haploid segregants; *leu2Δ* enabled selection for the 2-micron expression plasmid carrying *LEU2; ura3Δ* was used as a selectable marker during library construction; *pep4Δ*::*KanMX* reduced proteolytic activity to improve recombinant protein stability. In addition, the *MAT*-locus contains the genotype of either *MAT***a** or *MAT***α** and the *LYS2* locus, *LYS2* or *lys2Δ::URA3.* Second, was the diploid library of *hoΔ::HphMX/hoΔ::HphMX; MAT****a****/MAT****α***; *leu2Δ/leu2Δ; ura3Δ/ura3Δ; LYS2/lys2Δ::URA3 with a PEP4* locus genotype of either *pep4Δ::KanMX/pep4Δ::KanMX* or *PEP4/pep4Δ::KanMX.* These were transformed with expression plasmids for the production of an AP205 virus-like-particle protein (*Acinetobacter phage* AP205 coat protein (UniProt: Q9AZ42_9VIRU)) and SARS-CoV-2 spike protein fragment (YP_009724390.1, SARS-CoV-2 Receptor Binding Motif (RBM) fragment (residues 438–505) provided by Phenotypeca Ltd. Both were C-terminally fused to the fluorophore, mCherry from *Anaplasma marginale* (UniProt X5DSL3), which in the case of AP205 was via a linker sequence GSGGSGGSGPVTN. The whole-2-micron production plasmids were generated by Phenotypeca Ltd by in vivo homologous recombination in yeast using two linear DNA fragments with homology arms: one fragment containing the whole 2-micron backbone and one containing the recombinant expression cassette [29].

Strains from the libraries were grown in *B*uffered *M*inimal *M*edia with d-glucose (*D*extrose) (BMMD) supplemented with *C*omplete *S*upplement *M*ixture (CSM) without *leu*cine (-Leu) or *met*hionine (-Met) from Formedium (Catalogue No. DCS0549), to make media known as BMMD + CSM – Leu – Met. The medium comprised 0.17% yeast nitrogen base without amino acids or ammonium sulphate (Difco, Catalogue No. 233520), 0.5% ammonium sulphate (Sigma, Catalogue No. A4418), 0.69% citric acid (Sigma, Catalogue No. 27109), 2.243% sodium phosphate, dibasic dihydrate (Thermo Scientific, Catalogue No. 10782602), 2% D-glucose (Oxoid, Catalogue No. 10786973), 0.069% Complete Supplement Mixture dropout leucine and methionine (CSM – Leu – Met, Formedium, Catalogue No. DCS0549). To inhibit *MET17* promoter activity for the duration of batch cultivation, methionine (Sigma, Catalogue No. M9500) was added to 3 mM. Where necessary, the medium was solidified with 2% (w/v) agar (Oxoid, Catalogue No. 10351303). Yeast cultures (500 µL) were grown in 48-well microtiter plates (Greiner Bio-One) from single colonies at 30 °C, 200 rpm (50 mm orbit).

The yeast strain used for testing the *MET17* promoter activity and the FLP-mediated recombination efficiency assay (below) was the haploid laboratory *S. cerevisiae* strain BY4741 (*MAT***a**; *his3*Δ*1*; *leu2*Δ*0*; *met15*Δ*0*; *ura3*Δ*0*), maintained and grown in YNB broth comprising 0.69% yeast nitrogen base without amino acids (Formedium), 2% D-glucose or D-galactose, supplemented as required with amino acids or nucleotide bases, or in YPD broth comprising 1% yeast extract (Oxoid), 2% bacteriological peptone (Oxoid), 2% D-glucose. Where necessary, the medium was solidified with 2% (w/v) agar. Yeast cultures (10 mL) were grown in 50-mL Erlenmeyer flask from single colonies at 30 °C, 120 rpm (50 mm orbit).

Yeast transformation was performed by the lithium acetate/single-stranded carrier DNA/polyethylene glycol method [30]. Genomic DNA was extracted using the Wizard Genomic DNA Purification Kit (Promega) according to the manufacturer’s instructions. Appropriate integration of transformant DNA was checked by PCR and gel electrophoresis using standard procedures [31].

*E. coli* XL1-Blue electrocompetent cells were used for cloning and plasmid amplification. The cells were grown in Luria-Bertani (LB) medium containing 100 µg/mL ampicillin at 37 °C, 150 rpm (50 mm orbit). Where necessary, the medium was solidified with 2% (w/v) agar. Electroporation was carried out using a MicroPulser Electroporator (BIO-RAD) at 1.8 kV. Cells were recovered in 1 mL LB medium at 37 °C for one hour before spreading onto LB agar plates supplemented with 100 µg/mL carbenicillin.

### Ploidy test

The ploidy of selected strains from the libraries was determined by mating each strain with the test *S. cerevisiae* strains Y55-2369 (*MAT***α**; *ura2*Δ*1; tyr1*Δ*1*) and Y55-2370 (*MAT****a***; *ura2*Δ*1; tyr1*Δ*1*). Mating was performed by incubating each library strain with both test strains, respectively, in BMMD supplemented with 0.002% w/v leucine, using 48-well microtiter plates at 30 °C, 200 rpm (50 mm orbit). To assess growth, the Optical Density (OD) of the cultures was measured at 620 nm after three days. The ploidy was inferred based on the following criteria: the haploid strains grow only when co-cultured with a test strain of the opposite mating type, restoring prototrophy through complementation by mating; the diploids either fail to grow under both those conditions or grow independently, regardless of the presence of a Y55 strain.

### Quantification of mCherry expression and multi-generational production monitoring

mCherry fluorescence was measured at excitation wavelength 540/35 nm and emission wavelength 600/40 nm, while the OD was read at 620 nm using a Synergy HTX Multi-Mode Reader (BioTek). For monitoring over many generations, strains were cultivated in BMMD + CSM – Leu – Met medium, in 48-well microtiter plates 30 °C, 200 rpm and every 3 days, 2 µL of culture was transferred into a new 48-well microtiter plate containing 498 µL fresh medium. The fluorescence of the whole cultures with cells in suspension was measured every 3 days before each transfer for total mCherry fluorescence and OD_620_. No external fluorescent reference standard was used. Fluorescence values were therefore interpreted as relative fluorescence units (RFU), normalised to OD_620_, and compared across experiments performed under matched instrument settings. The percentage change in mCherry fluorescence over the duration of the experiment was calculated by the following equation:

$$ \:\frac{{\begin{array}{*{20}c} {Initial\:mCherry} \\ {fluorescence} \\ \end{array} \:\: - \:\begin{array}{*{20}c} {mCherry\:fluoresnce\:at\:the} \\ {end\:of\:generaiton} \\ \end{array} \:}}{{\begin{array}{*{20}c} {Initial\:mCherry} \\ {fluorescence} \\ \end{array} }} \times \:100\% $$

### Plasmid purification from yeast and whole plasmid sequencing

The production plasmids were purified directly from yeast cells, according to the protocol from Singh and Weil [32]. Yeast cultures (100 mL) were grown in BMMD + CSM – Leu – Met medium in 500-mL Erlenmeyer flasks from single colonies at 30 °C, 120 rpm (50 mm orbit) overnight. The Qiagen Plasmid Miniprep Kit (Qiagen, Catalogue No. 27106) was used for plasmid purification, according to the manufacturer’s instructions with minor modifications. Briefly, the overnight cultures were pelleted by centrifugation (1000×*g*, 5 min) and resuspended in 2 mL Buffer P1 by vortexing. The cell suspension was mixed with 80 µL of 75 units/ µL lyticase from *Arthrobacter luteus* (Sigma, Catalogue No. L2524) and incubated at 37 °C for 2 h to digest the cell wall. Cells were lysed by adding 2 mL Buffer P2 and mixed by gently inverting followed by 10 min incubation at room temperature. 2.8 mL Buffer N3 was then added to the lysate and mixed by gently inverting, then incubated for 30 min on ice. The supernatant was collected by centrifugation (13,000×*g*, 10 min) at 4 °C and applied to three Qiagen plasmid miniprep columns. The columns were washed once with 500 µL Buffer PB and then washed twice with 750 µL Buffer PE. Plasmid DNA was eluted by adding 30 µL nuclease-free water to the column, incubating at room temperature for 5 min and centrifuging at 13,000×*g* for 1 min. The plasmid concentration was quantified using the Qubit dsDNA assay kit (Invitrogen, Catalogue No. Q32851) and sequenced by Oxford Nanopore technique (Full Circle Labs). The expected plasmid size was approximately 9.9 kb. Across the sequenced plasmid samples, 145 to 628 reads were obtained per sample, corresponding to an estimated raw sequencing coverage of approximately 80× to 300× per sample. Mean per-read quality scores ranged from Q15.9 to Q17.8. Read N50 values ranged from 9794 bp to 9885 bp, consistent with near full-length plasmid reads. Plasmid sequences from evolved samples were aligned against the corresponding 10th -generation plasmid sequence as the reference using ClustalW. Sequence variants were identified from differences in these alignments and manually inspected.

### Total DNA extraction and droplet digital PCR (ddPCR)

Total DNA was extracted from yeast cells using the DNeasy Blood & Tissue Kit (Qiagen, Catalogue No. 69504), following the manufacturer’s supplementary protocol for yeast DNA purification. The DNA concentration was measured using the Qubit dsDNA assay kit (Invitrogen) and diluted to 0.1 ng/ µL. For plasmid copy number quantification, 2 µL of diluted DNA (0.1 ng/ µL) was used as a template. Reactions were prepared in a 96-well QIAcuity Nanoplate 8.5k (Qiagen, Catalogue No. 250021) with a total reaction volume of 12 µL, using the QIAcuity EG PCR kit (Qiagen, Catalogue No. 250112) according to the manufacturer’s instructions. Primers used for ddPCR were listed in Table S1. The reaction was run on the QIAcuity Digital PCR system (Qiagen). The *S. cerevisiae ACT1* housekeeping gene was used as a reference gene, and a plasmid-specific noncoding sequence was used as the target for quantification. The plasmid concentration obtained (copies/µL) was normalised to *ACT1* to determine plasmid copy number per genome.

### Promoter activity assay

To test the *MET17* promoter activity, reporter plasmids were constructed. Primers used for the construction were listed in Table S1. The wild-type *MET17* promoter and mCherry coding sequence were amplified from the production plasmids extracted directly from the yeast at the 10th generation. The mutated *MET17* promoter was amplified from the production plasmid carrying the identified mutation, and the *CYC1* terminator sequence (*tCYC1*) was amplified from plasmid pCM190 (Euroscarf). *pMET17* (wild-type or mutated), *mCherry* and *tCYC1* sequences were ligated between the *Sac*I and *Sal*I sites in pRS315 (ATCC) (to give pRS315-*pMET17*(wild-type)-*mCherry-tCYC1* and pRS315-*pMET17*(mutated)-*mCherry-tCYC1* plasmid constructs) using a HiFi DNA assembly cloning kit (New England Biolabs, NEB, Catalogue No. E5520) according to the manufacturer’s instructions. The constructed reporter plasmids were used to transform *S. cerevisiae* BY4741. The transformants were selected on YNB agar lacking leucine and arising colonies were grown overnight in YNB broth lacking leucine. Aliquots (500 µL) of the overnight culture were transferred to 48-well microtiter plates for mCherry measurement as described earlier.

### *FLP*-mediated recombination efficiency assay

Primers used for the construction were listed in Table S1. To assess the efficiency of FLP-mediated recombination via *FRT* sequences, *S. cerevisiae* strain BY4741 was engineered to express the FLP recombinase by integration of the *FLP1* gene under the control of the *GAL1* promoter at the *HO* locus, using *HIS3MX6* as a selection marker. To do this, the *FLP1* open reading frame (ORF) was amplified from the production plasmid extracted directly from the yeast at the 10th generation and a *tCYC1* was amplified from plasmid pCM190. The *FLP* ORF and *tCYC1* were assembled between the *Sal*I and *Hind*III sites in pFA6a-*His3MX6-pGAL1* [33] to give pFA6a-*His3MX6-pGAL1-FLP-tCYC1* construct, via Gibson assembly using a HiFi DNA assembly cloning kit (NEB, Catalogue No. E5520S) according to the manufacturer’s instructions. The *His3MX6-pGAL1-FLP-tCYC1* fragment was amplified by PCR with 50 bp homology arms to the *HO* locus and then used for transformation into *S. cerevisiae* BY4741. Transformants (*ho*Δ::*His3MX6-pGAL1-FLP-tCYC1*) were selected in YNB agar lacking histidine.

A *KanMX* resistance cassette was amplified from pFA6A-*KanMX6* [34], using primers containing *Sac*I and *Sal*I restriction sites together with *FRT* site variants to be tested flanking the *KanMX* cassette in the same orientation. The *FRT-KanMX-FRT* fragments were assembled into the pRS315 vector between the *Sac*I and *Sal*I sites using T4 DNA ligase (NEB), according to the manufacturer’s instructions. The resulting plasmid constructs were transformed into the engineered BY4741 strain expressing *FLP1* recombinase (*ho*Δ::*His3MX6-pGAL1-FLP1-tCYC1*). Transformants were selected in YNB agar lacking histidine and leucine. The transformants with the plasmid constructs were first precultured overnight in YNB broth minus histidine and leucine, using 2% D-glucose as the carbon source. Cells (OD_600_ ~ 1.0) were harvested by centrifugation (1000 x *g*, 2 min) and resuspended to OD_600_ ~ 0.1 in 10 mL YNB medium containing 2% galactose to induce *FLP1* expression. Induced cultures were incubated at 30 °C, 120 rpm (50 mm orbit), with serial passaging (diluting overnight cultures at OD_600_ ~ 3–4 to OD_600_ ~ 0.1 in fresh selective YNB medium with 2% galactose as carbon source) for approximately 50 generations to maintain continuous *FLP1* induction. After 50 generations, 100 µL of diluted cultures (OD_600_ ~ 1 × 10^− 4^) were plated on YPD agar (non-selective) and YPD agar containing 200 µg/mL G418 (selective for non-recombined plasmids). Recombination efficiency was calculated following the equation:

$$\:\frac{(\mathrm{t}\mathrm{h}\mathrm{e}\:\mathrm{n}\mathrm{u}\mathrm{m}\mathrm{b}\mathrm{e}\mathrm{r}\:\mathrm{o}\mathrm{f}\:\mathrm{c}\mathrm{o}\mathrm{l}\mathrm{o}\mathrm{n}\mathrm{i}\mathrm{e}\mathrm{s}\:\mathrm{o}\mathrm{n}\:\mathrm{Y}\mathrm{P}\mathrm{D}\:-\:\mathrm{t}\mathrm{h}\mathrm{e}\:\mathrm{n}\mathrm{u}\mathrm{m}\mathrm{b}\mathrm{e}\mathrm{r}\:\mathrm{o}\mathrm{f}\:\mathrm{c}\mathrm{o}\mathrm{l}\mathrm{o}\mathrm{n}\mathrm{i}\mathrm{e}\mathrm{s}\:\mathrm{o}\mathrm{n}\:\mathrm{Y}\mathrm{P}\mathrm{D}\:\mathrm{c}\mathrm{o}\mathrm{n}\mathrm{t}\mathrm{a}\mathrm{i}\mathrm{n}\mathrm{i}\mathrm{n}\mathrm{g}\:\mathrm{G}418)}{\mathrm{t}\mathrm{h}\mathrm{e}\:\mathrm{n}\mathrm{u}\mathrm{m}\mathrm{b}\mathrm{e}\mathrm{r}\:\mathrm{o}\mathrm{f}\:\mathrm{c}\mathrm{o}\mathrm{l}\mathrm{o}\mathrm{n}\mathrm{i}\mathrm{e}\mathrm{s}\:\mathrm{o}\mathrm{n}\:\mathrm{Y}\mathrm{P}\mathrm{D}}$$

### Growth measurement

Growth of yeasts was followed using a Synergy HTX Multi-Mode Reader. In brief, 5 µL of 72 h culture in BMMD + CSM – Leu – Met medium was transferred to wells of 48-well microtiter plates containing 495 µL of BMMD + CSM – Leu – Met medium. The OD_620_ was measured every 30 min during continuous linear shaking (1096 cycles min^− 1^, 1 mm orbit) at 30 °C for 72 h. The specific growth rates were calculated during the exponential growth phase using OD measurements taken over a 3-h time interval.

### Flow cytometry

For the SARS-CoV-2-mCherry fusion protein, intracellular mCherry fluorescence measured by flow cytometry was interpreted as a relative proxy for intracellular expression or production-associated state, not as a direct measure of total protein yield. Cells were diluted to OD_620_ ~0.5 in PBS. Flow cytometric analysis was performed using a CytoFLEX S Flow Cytometer (Beckman Coulter) fitted with a blue (488 nm) laser. Forward scatter (FSC-A) was used to assess cell size. Cells were first gated using forward scatter and side scatter parameters to exclude debris and events with abnormal scatter profiles. Where applicable, single cells were further selected using FSC-A and FSC-H to minimise doublet events. mCherry fluorescence was then analysed for the gated cell population and normalised to cell size using FSC-A. For classification of fluorescence-defined subpopulations, the non-fluorescent threshold was defined using the empty-vector control strain, which represented background autofluorescence. The thresholds separating low-fluorescence and high-fluorescence subpopulations were then set based on the tripartite fluorescence distribution observed in the corresponding 10th-generation initial cultures. The same thresholds were applied consistently to the matched evolved cultures to compare changes in the proportions of non-fluorescent, low-fluorescence and high-fluorescence cells. mCherry fluorescence was detected using B585 (585/42 emission filter) channels. Cell sorting was conducted using an Astrios Cell Sorter (Beckman Coulter). After setting appropriate gates, 1.5 × 10^4^ cells were sorted into sterile PBS for subsequent experiments.

### Sodium dodecyl sulphate polyacrylamide gel electrophoresis (SDS-PAGE)

Retransformants harbouring single-type plasmids were cultured (10 mL) in BMMD + 2x CSM – Leu – Met medium (2× CSM, 0.138% w/v) in 50 mL Erlenmeyer flasks at 30 °C, 120 rpm (50 mm orbit) for 3 days. Cultures (1 mL) were then centrifuged at 10,000×*g* for 2 min, and 500 µL of supernatant was collected. A 32.5 µL aliquot of each supernatant sample was mixed with 5 µL of NuPAGE™ Sample Reducing Agent (10x, ThermoFisher, Catalogue No. NP0009) and 12.5 µL of NuPAGE™ LDS Sample Loading Buffer (4x, ThermoFisher, Catalogue No. NP0007). The mixtures were heated at 80 °C for 10 min to denature proteins. Prepared samples (30 µL) were then loaded onto pre-cast NuPAGE™ Bis-Tris gel (4–12%, ThermoFisher, Catalogue No. NP0322B0X) using 1x MES SDS Running buffer, with 500 µL of NuPAGE™ Antioxidant (ThermoFisher, Catalogue No. NP0005) added to the inner chamber. 5 µL of PageRuler™ Prestained Protein Ladder (10 to 180 kDa, ThermoFisher, Catalogue No. 26616) was used as a molecular weight marker. SDS-PAGE was run at 200 V for 35 min. Once the dye front had fully migrated, the gel was removed and stained using GelCode™ Blue Safe Protein Stain (ThermoFisher, Catalogue No. 24594) according to the manufacturer’s instructions.

### Statistical analyses

Data were typically expressed as the mean of at least three biological replicates ± standard error of the mean (SEM). Statistical significance was determined by analysis with one-way or two-way ANOVA or *t-*test, using GraphPad Prism 9 software. A p-value less than 0.05 (*p* < 0.05) was considered statistically significant. *p* < 0.05(*), *p* < 0.01(**), *p* < 0.001(***), *p* < 0.0001(****).

## Results

### Long-term recombinant protein expression results in diversification and selection

**Fig. 1:**
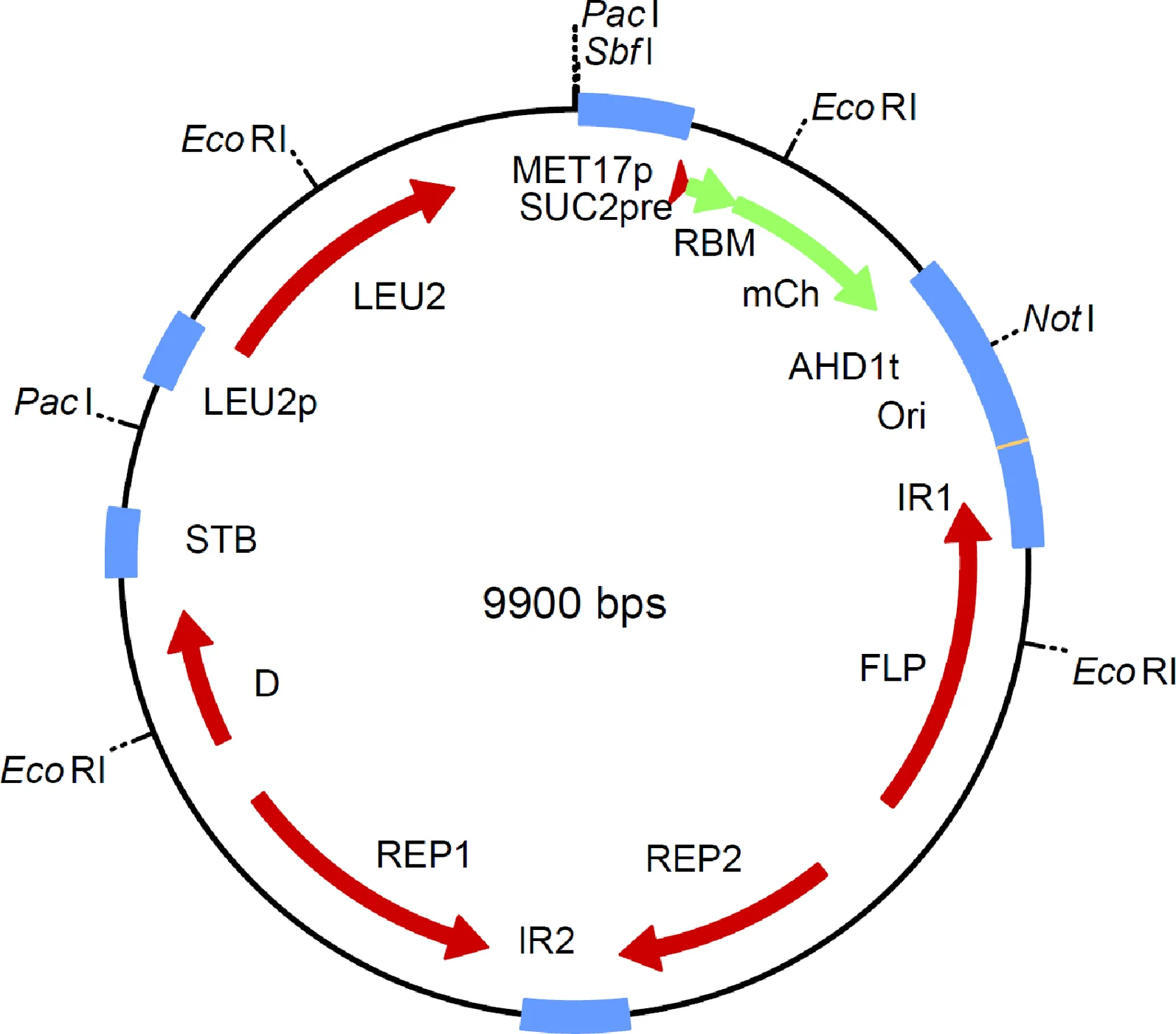
Schematic map of the whole-2-micron expression plasmid for secretion of the SARS-CoV-2 spike fragment mCherry fusion protein. The production plasmid is based on the endogenous *S. cerevisiae* 2-micron plasmid and contains the whole 2-micron backbone, including the replication and partitioning genes *REP1*,* REP2* and *RAF1 (D)*, as well as the *FLP1* recombinase gene. Inverted repeat regions, IR1 and IR2, and the stability locus, *STB*, are annotated. The *LEU2* gene serves as a selection marker. Heterologous DNA comprising the *LEU2* gene and the SARS-CoV-2 spike fragment mCherry fusion protein was inserted at a *Sna*BI site downstream of the *STB* locus in the native 2-micron plasmid. The SARS-CoV-2 spike fragment (RBM) is under the control of the *MET17* promoter (MET17p) and is C-terminally tagged with mCherry (mCh) to enable quantitative fluorescence-based monitoring of expression

This study used genetically diverse haploid and diploid yeast libraries generated through advanced multi-generational breeding [35]. These libraries were derived from four distinct clean parental lineages (i.e. North American, Sake, West African and European/Wine). Each parental strain had been engineered to enhance recombinant product yield through reduced proteolysis by deletion of the *PEP4* gene encoding the master protease proteinase A [36] and transformed with high copy, whole-2-micron based plasmids expressing either an intracellular AP205 virus-like-particle (VLP) protein or a secreted product SARS-CoV-2 spike protein fragment from sequences inserted at a *Sna*BI site downstream from the stability locus binding (*STB*) region (which mediates interaction with host proteins to ensure plasmid partitioning) in the 2-micron plasmid (Fig. 1). All strains had previously been cured of the native 2-micron plasmid to prevent potential interactions with the introduced expression plasmid. The expression plasmids were assembled by homologous recombination in vivo during host cell transformation with two linear DNA fragments, one comprising the whole 2-micron sequence and the other comprising the expression construct for the recombinant protein of interest [37]. Expression was under the control of the *MET17* promoter, which is repressible by adding excess extracellular methionine above 0.05 mM, allowing for regulated expression [38, 39]. Both the AP205 VLP protein and the SARS-CoV-2 spike protein were tagged with C-terminal mCherry. Hereafter, they are referred to as AP205 VLP-mCherry fusion and SARS-CoV-2-mCherry fusion, respectively.

To investigate whether high expression would accelerate diversification and selection in the yeast cell populations, we first screened and selected 36 strains randomly from both the haploid and diploid libraries (i.e. 72 strains in total) expressing either AP205 VLP-mCherry fusion or SARS-CoV-2-mCherry fusion. Both ploidy types were included to assess whether diploids, often considered more robust and genetically stable than haploids [40, 41], displayed greater production stability. mCherry fluorescence was quantified for all strains to assess their level of production (Figure S1). The haploid library had been generated by Phenotypeca Ltd through sporulation of the diploid library, followed by diethyl ether treatment to selectively remove non-sporulated diploid cells and zymolyase treatment, with plating out to isolate colonies from individual spores [42]. Since this method does not ensure complete removal of diploids, ploidy testing was also conducted on selected strains from the haploid library to verify their haploid status.

**Fig. 2:**
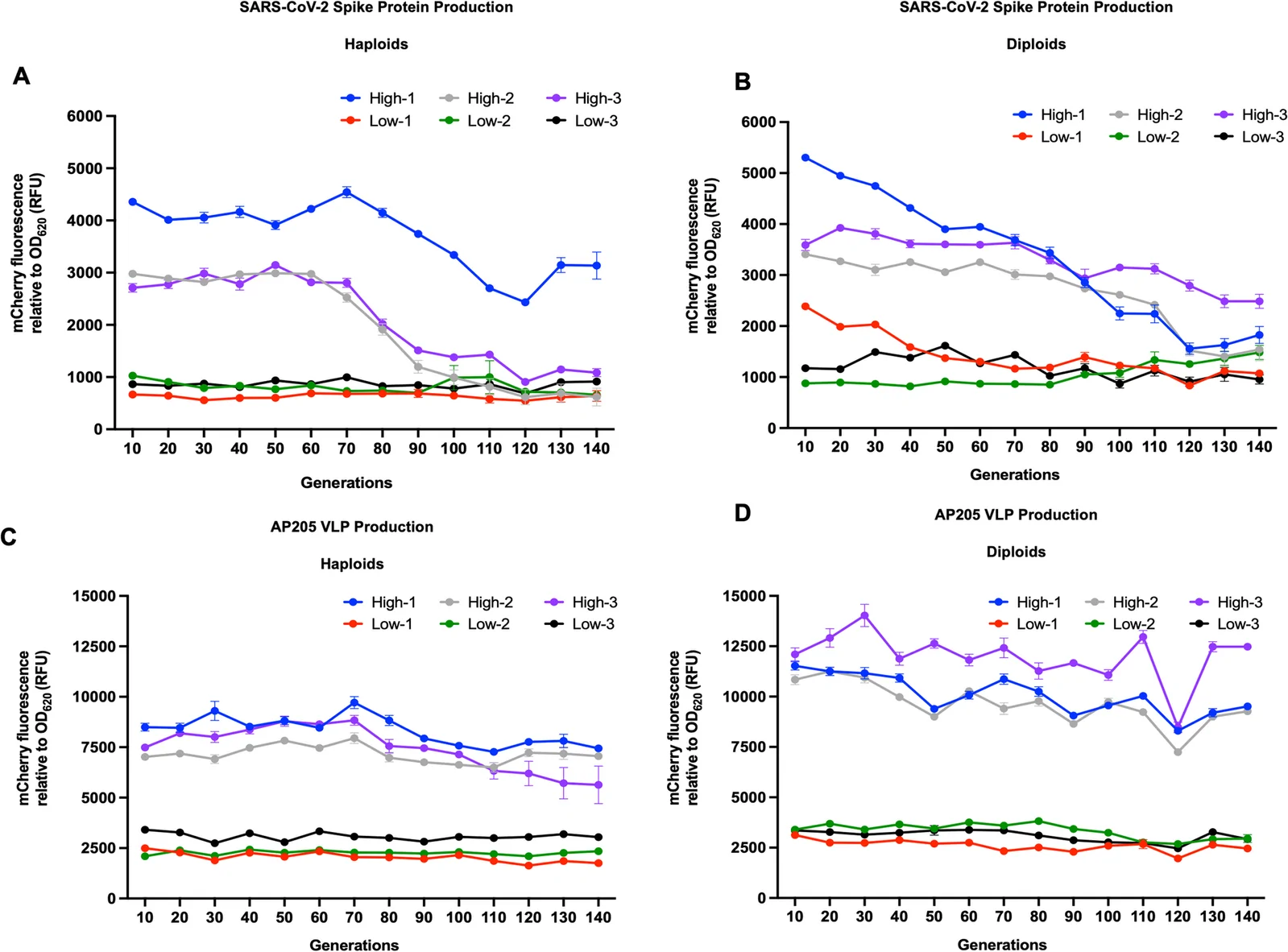
mCherry levels over multiple generations for selected strain candidates from genetically diverse libraries secreting a SARS-CoV-2 spike protein fragment or intracellular AP205 VLP protein. mCherry RFU (normalised to OD_620_) was measured every ~ 10 generations during sequential subculture into fresh medium every 3 days. mCherry fluorescence in three high-fluorescence strains (designated High-1, High-2, High-3) and three low-fluorescence strains (Low-1, Low-2, Low-3) from the haploid (**A**, **C**) or diploid (**B**, **D**) libraries for the SARS-CoV-2 spike protein fragment (**A**, **B**) or the AP205 VLP protein (**C**, **D**). Each data point shown is the mean value ± SEM from eight biological replicates (i.e. eight clones derived from a single strain stock)

To assess the performance of simulated long-term bioproduction, three high-fluorescence strains and three low-fluorescence strains from each library were selected for further analysis. mCherry fluorescence of the cultures (8 clones for each strain, where a clone of a strain refers to a clonal colony derived from a single cell of that strain) was monitored every ~ 10 generations during sequential batch cultivation in 48-well microtiter plates (every 3 days, aliquots of culture were subcultured to OD_620_ ~0.05 in fresh medium). Fluorescence levels were first recorded after the initial 3-day growth period (i.e. after ~ 10 generations), as this allowed cells to reach the stationary phase and reflected their early expression capacity. At this point, approximately fourfold to sixfold differences in both AP205 VLP-mCherry fusion and SARS-CoV-2-mCherry fusion fluorescence levels were observed between the highest and lowest fluorescent strains among both haploid and diploid strains (Fig. 2). Both the haploid and diploid strains that showed high-fluorescence for the secreted SARS-CoV-2-mCherry fusion exhibited a decline in fluorescence over time (Fig. 2A and B), whereas those expressing intracellular AP205 VLP-mCherry fusion maintained comparatively stable fluorescence over 140 generations (Fig. 2C and D), suggesting that AP205 VLP-mCherry production did not provide significant stress to the strains. The decline in SARS-CoV-2-mCherry production was particularly evident in High-2 and High-3 strains from the haploid library (Fig. 2A) and in High-1 and High-2 strains from the diploid library (Fig. 2B). Low-fluorescence strains for both products showed stable fluorescence throughout the cultivation period, suggesting a potential trade-off between initial production yield and long-term stability. The SARS-CoV-2 protein is a fragment of the spike protein containing three disulphide bonds, which was therefore expected to represent a potentially more challenging folding substrate for the endoplasmic reticulum in *S. cerevisiae*. However, the different stability profiles of the SARS-CoV-2-mCherry and AP205 VLP-mCherry systems may reflect multiple factors, including secretion burden, folding complexity, disulphide bond formation and potential toxicity. Given the faster fluorescence decline observed for strains expressing the SARS-CoV-2-mCherry fusion than those expressing the AP205 VLP-mCherry fusion (Fig. 2), we chose to focus on the strains expressing the former to explore the relationship between production burden and evolutionary dynamics.

**Fig. 3:**
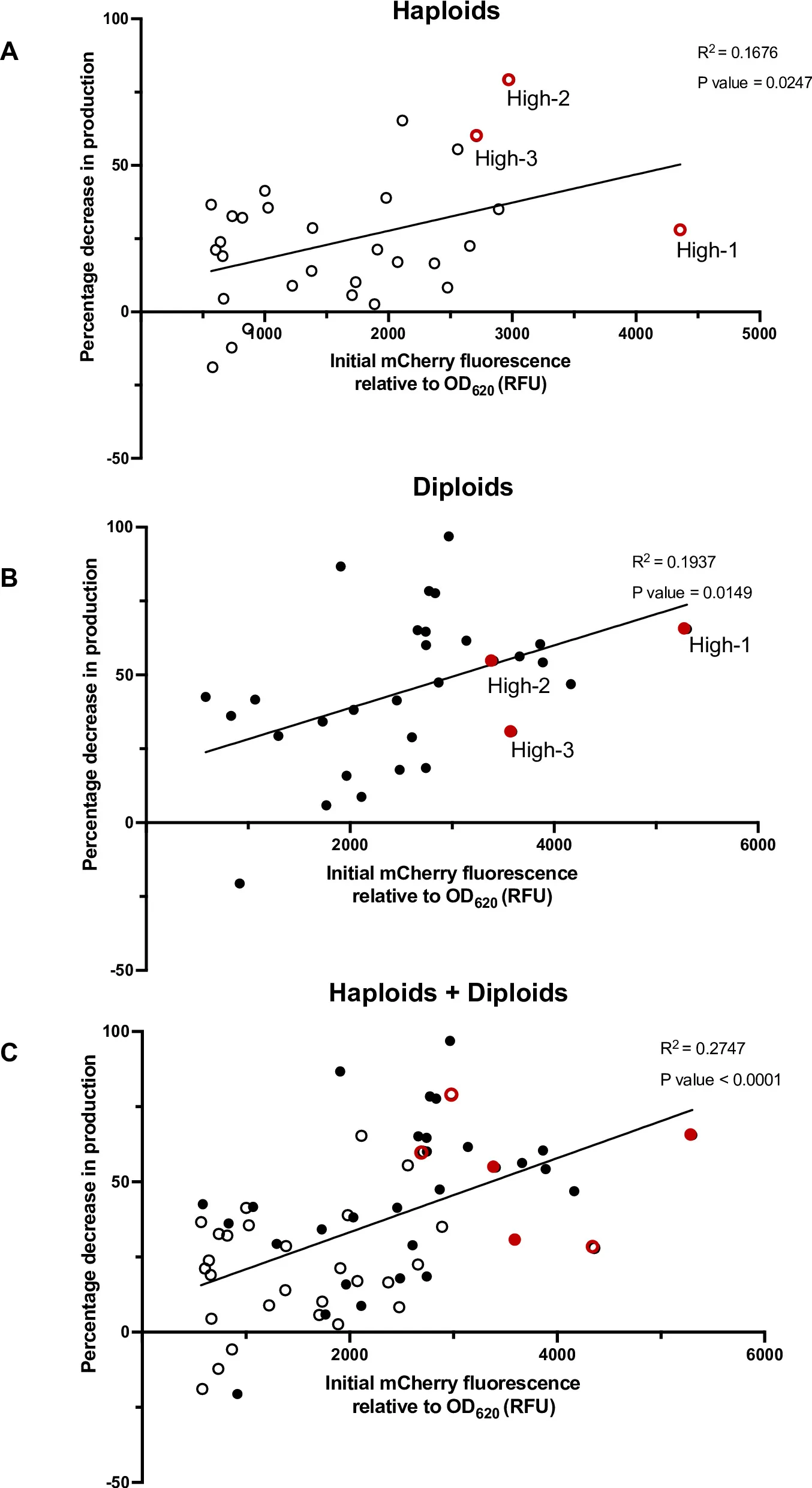
Relationship between initial SARS-CoV-2-mCherry fusion protein expression level and fluorescence decline after 140 generations. Strains were cultured in 48-well plates for 72 h in BMMD + CSM – Leu – Met medium. **A**, **B** Data for 30 strains (24 randomly selected plus the six strains from Fig. 2) from each of the diploid (**A**) and haploid (**B**) libraries. **C** Combined data from all 60 strains. Each data point is the mean of eight biological replicates, where diploids are shown as closed circles and haploids as open circles. The three selected high-fluorescence strains from each library are highlighted in red. Fitted lines represent simple linear regression. Initial fluorescence-based expression was positively associated with subsequent fluorescence decline: **A** R² = 0.168, Pearson r = 0.409, 95% CI 0.058 to 0.671, p = 0.0247, n = 30; **B** R² = 0.194, Pearson r = 0.440, 95% CI 0.095 to 0.691, p = 0.0149, n = 30; and **C** R² = 0.274, Pearson r = 0.523, 95% CI 0.311 to 0.686, p < 0.0001, n = 60

To test whether there is a correlation between the initial production level and production stability, we cultivated 24 additional transformants that represented a broad range of expression levels from both the haploid and diploid libraries expressing SARS-CoV-2-mCherry fusion protein and monitored their mCherry fluorescence over 140 generations. To quantify production stability, the percentage decrease in mCherry fluorescence over 140 generations was calculated using data from both the 24 additional strains and the six previously analysed high- and low- fluorescent strains. The results showed a moderate negative correlation between initial fluorescence level and the extent of fluorescence decline over 140 generations: the strains with higher initial fluorescence levels generally experienced the greater declines over time (Fig. 3). This trend was observed in both haploid and diploid backgrounds. Although the results suggested that higher fluorescence as a proxy for expression imposes more burden and contributes to instability, the relationship was not absolute, suggesting that the relative fluorescence level was not the only factor determining long-term fluorescence decline.

Certain strains were clear outliers to the general trend seen above, linking higher initial fluorescence levels with increased expression stability (Fig. 3). For example, the High-1 strain from the haploid library, which showed the highest initial production level among the 30 haploid strains tested, maintained moderate to high production throughout the 140 generations (Figs. 2A and Fig. 3A). Among the selected diploid strains, the High-2 and High-3 diploid strains exhibited comparable initial production levels, but the High-2 diploid strain experienced an approximate 50% decrease in production over 140 generations, whereas the High-3 diploid strain showed only an approximate 25% decrease (Fig. 3B). These results highlight the functional diversity present within the starting strain pool, where some rare genotypes may possess both high expression capacity and resilience to production-associated stresses. This suggests that since the initial fluorescence level does not appear to be the sole factor influencing fluorescence decline, high-fluorescence strains that provide more stable expression over time can be selected from genetically diverse libraries.

### Repression and re-induction reveal partial reversibility of expression decline

**Fig. 4:**
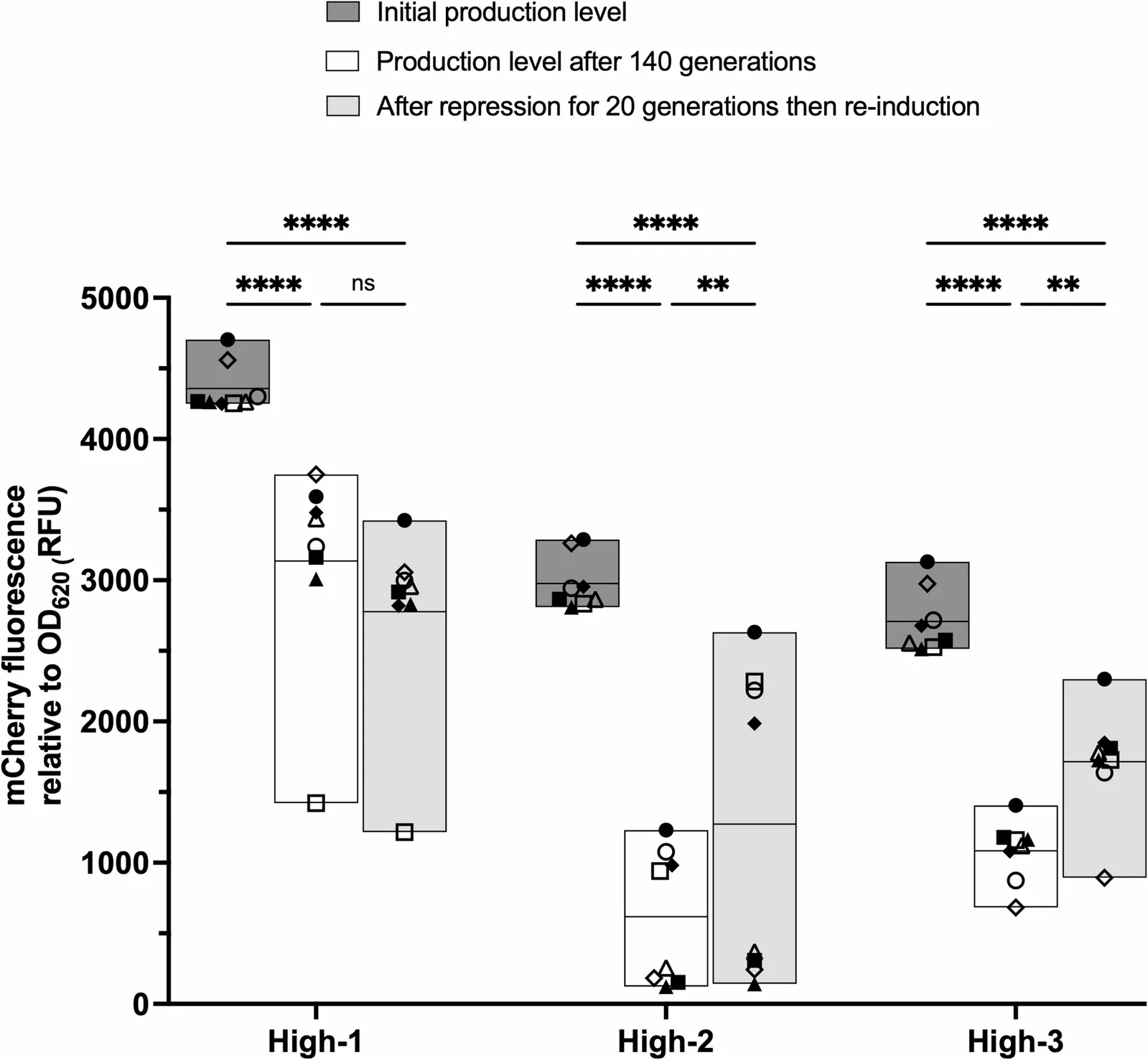
Expression of the SARS-CoV-2-mCherry fusion protein from cultures at the 140th generation following repression of production and re-induction. Eight independently replicated cultures of each haploid strain (High-1, High-2, High-3) previously passaged for 140 generations under production-inducing conditions were grown in 48-well microtiter plates with 500 µL BMMD + CSM – Leu + Met medium (i.e. production-repressing condition) for approximately 20 generations (two sequential 3-day passages of ~ 10 generations each) to repress MET17 promoter activity. After this repression phase, cultures were transferred into 500 µL BMMD + CSM – Leu – Met medium to re-induce production, and mCherry levels were measured and normalised to OD_620_. Initial production (measured after 10 generations) and production after 140 generations from the long-term cultivation experiment are shown as controls (see Fig. 2A). Symbols indicate the eight replicate cultures originally established for each strain in the long-term production experiment: culture 1 (●), culture 2 (○), culture 3 (■), culture 4 (□), culture 5 (▲), culture 6 (△), culture 7 (◆), culture 8 (◇). The floating bars represent the full data range (min to max); the internal horizontal line indicates the mean. Statistical significance (Two-way ANOVA): p < 0.01 (**), p < 0.0001 (****), not significant (ns)

To investigate declines in SARS-CoV-2-mCherry production, we focused on haploid strains because their single set of chromosomes simplifies genetic interpretation and analysis. Specifically, the haploid High-1, High-2 and High-3 strains from the long-term production experiment (Fig. 2A) were examined. We hypothesised that if the fluorescence decline is primarily driven by non-genetic factors, such as cell-to-cell phenotypic heterogeneity, which can generate subpopulations with differing resilience to arising stresses [43], then temporarily repressing expression could allow cells to recover from stress and revert to a production-competent state once the production burden was relieved. To test this, the eight replicate cultures of each starting strain (maintained under production-inducing conditions for 140 generations) were grown in BMMD + CSM – Leu + Met medium for approximately 20 generations to repress expression from the *MET17* promoter. This repression phase was intended to reduce production-associated burden and allow cells to recover. After this repression phase, cells were washed to remove excess methionine and then transferred back into BMMD + CSM – Leu – Met medium to re-induce production, and SARS-CoV-2-mCherry production was measured (Fig. 4). For the High-1 strain, no significant increase in fluorescence was observed after the repression and re-induction, with fluorescence levels remaining comparable to those at the 140th generation. This suggests that in High-1 cultures, the reduced-fluorescence state was stable and unresponsive to short-term changes in induction conditions. In contrast, both High-2 and High-3 strains displayed significant increases in average fluorescence across their eight replicate cultures after the repression phase compared to their fluorescence levels at the 140th generation. Nevertheless, even after repression and re-induction, production remained significantly lower than the initial levels. Notably, there were marked differences in response between High-2 cultures. Specifically, cultures 1, 2, 4 and 7 showed evident improvement in fluorescence after repression and re-induction, whereas cultures 3, 5, 6 and 8 remained at low fluorescence levels (Fig. 4). This pattern indicates that over 140 generations, replicate cultures of the same starting strain followed different phenotypic and/or genetic outcomes. Some cultures became unresponsive to repression and re-induction, while others retained partial reversibility, suggesting that non-genetic physiological states and more stable genetic or plasmid-associated changes may both contribute to fluorescence decline, although the timing of the emergence of stable plasmid variants was not directly determined.

### Single-cell analysis shows non-genetic heterogeneity in intracellular mCherry fluorescence

**Fig. 5:**
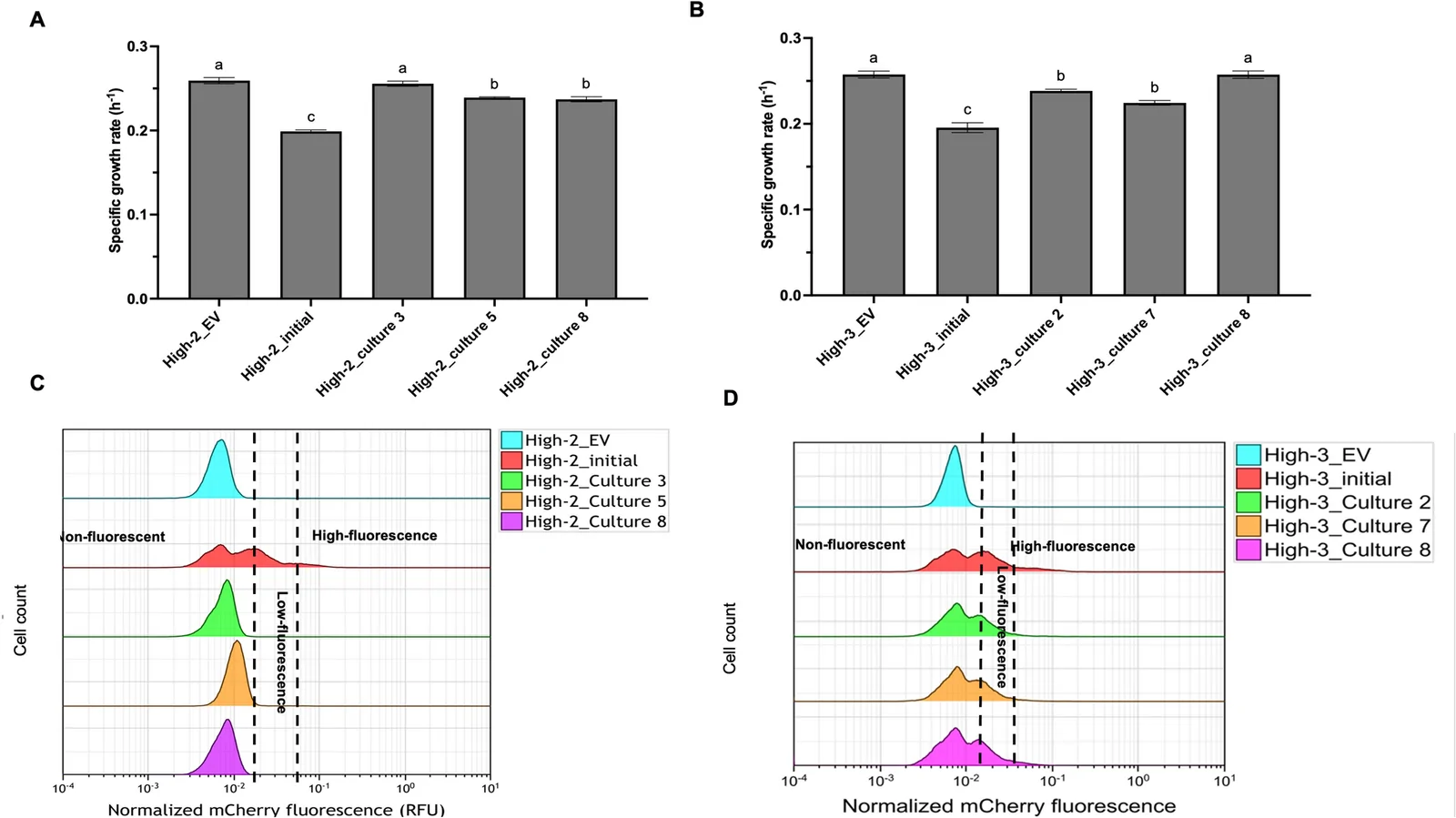
Growth rates and single-cell mCherry fluorescence profiles of High-2 and High-3 cultures for SARS-CoV-2-mCherry expression. High-2 and High-3 strains from the 10th generation were cured of plasmid, transformed with empty vector (EV) and used as controls. **A**, **B** Comparison of specific growth rates of High-2 (**A**) and High-3 (**B**) strains at early (10th generation) and late (140th generation) stages. Cells were cultured in sequential batch culture in BMMD + CSM – Leu – Met medium. Specific growth rates were calculated during the exponential growth phase using OD measurements taken over a 3-hour interval. Specific growth rates of cultures at the 140th generation are presented as mean values ± SEM of four biological replicates. Means with different letters are significantly different, as determined by ordinary one-way ANOVA, *p* < 0.05. **C**, **D** Flow cytometric analysis of intracellular mCherry fluorescence in High-2 (**C**) and High-3 (**D**) cultures. Single-cell mCherry intensity was analysed for initial cultures at the 10th generation (High-2_initial and High-3_initial), and the lowest-fluorescence cultures were selected at the 140th generation (High-2_Culture 3, 5 and 8, and High-3_Culture 2, 7 and 8). Dashed vertical lines indicate approximate thresholds distinguishing non-fluorescent, low- and high-fluorescence subpopulations. Each fluorescence distribution represents 10,000 individual cells, with mCherry fluorescence values normalised to cell size, FSC-A

The repression and re-induction experiment indicated that the fluorescence decline was partly reversible (non-heritable), suggesting a contribution from non-genetic factors. To explore this further, we examined intracellular mCherry fluorescence at the single-cell level, as individual cells in an isogenic population may differ in gene expression, metabolic activity and stress tolerance [44]. We hypothesised that cultures at the initial high-total-fluorescence stage (i.e. 10th generation) would exhibit broader distributions of intracellular mCherry fluorescence phenotypes than those at the 140th generation, because low-fluorescence cell subpopulations are likely to have a fitness advantage and become enriched over time. This was tested with the haploid High-2 and High-3 strains, as these showed the most significant fluorescence declines and marked differences between cultures at the 140th generation after repression and re-induction (Figs. 2A and Fig. 4). For each strain, three cultures with the lowest total fluorescence at the 140th generation were selected for detailed study (High-2 cultures 3, 5, and 8 and High-3 cultures 2, 7 and 8). Specific growth rates at the early high-fluorescence stage (i.e. 10th generation) were significantly lower than those of the evolved low-fluorescence cultures (i.e. 140th generation), consistent with a growth advantage associated with reduced fluorescence or reduced expression-associated burden (Fig. 5A and B). SARS-CoV-2-mCherry fusion protein expression was compared in 10,000 individual cells from cultures at the 10th generation (i.e. High-2_initial and High-3_initial) and from the selected cultures at the 140th generation (i.e. High-2_3, 5 and 8 and High-3_2, 7 and 8) (Fig. 5C and D). Cells were analysed during exponential growth phase to minimise confounding effects associated with stationary phase, such as reduced protein synthesis or extracellular accumulation [45, 46]. As the SARS-CoV-2-mCherry fusion protein was secreted, the fluorescence signal measured by flow cytometry cannot be interpreted as a direct measure of total recombinant protein production. Instead, the signal represents the intracellular mCherry-containing fraction present at the time of analysis, which might be influenced by expression level, secretion efficiency, intracellular retention, degradation, fluorescent protein maturation and dilution by growth. Therefore, single-cell fluorescence was interpreted as a relative proxy for intracellular expression state or production-associated state, under the assumption that mCherry maturation, degradation, dilution by growth and fluorescence per mature mCherry molecule were broadly comparable across the conditions being compared [47]. At the 10th generation, both High-2 and High-3 populations displayed broad, multimodal fluorescence distributions with three apparent subpopulations: non-fluorescent cells (fluorescence comparable to that of cells expressing the empty vector), low-fluorescence cells (intermediate fluorescence), and high-fluorescence cells (elevated fluorescence). The high-fluorescence subpopulation comprised only a small fraction of the total population (~ 10%), whereas most cells fell within the low-fluorescence or non-fluorescent ranges, indicating substantial cell-to-cell heterogeneity in intracellular mCherry fluorescence during the early high-total-fluorescence stage.

In contrast to the early high-total-fluorescence cultures, cells from the lowest-fluorescence cultures at the 140th generation showed markedly narrower fluorescence distributions, mainly within the non-fluorescent or low-fluorescence ranges (Fig. 5C and D). High-fluorescence cells were nearly absent, with fewer than 0.1% showing fluorescence comparable to that of the high-fluorescence subpopulation in the initial cultures. Consistent with this, the coefficient of variation (CV) in mCherry fluorescence was significantly lower in these cultures (Figure S2), indicating more compact distributions. These results indicate that higher fluorescence is associated with greater expression heterogeneity within the population, whereas low-fluorescence states are associated with reduced heterogeneity and enrichment of non-fluorescent or low-fluorescence cells.

To substantiate that the three subpopulations observed at the 10th generation were due to phenotypic variation rather than genetic differences, we used fluorescence-activated cell sorting (FACS) to isolate ~ 15,000 cells from each of the non-, low- and high-fluorescence subpopulations and regrew them in expression medium for three days. After regrowth, all sorted subpopulations showed mean expression levels similar to the original unsorted population (Figure S3), indicating that the fluorescence states were transient, i.e., non-inherited. With flow cytometry, it was also evident that each regrown subpopulation re-established a broad, multimodal fluorescence profile resembling the original cultures (Figure S4A). Post-sort reanalysis verified accurate gating and enrichment (Figure S4B). These results support that the observed subpopulations represent reversible, non-genetic phenotypic heterogeneity.

### Plasmid-encoded mutations explain stable low-fluorescence phenotypes in selected evolved isolates

Besides non-genetic factors, the results in Fig. 4 indicated genetic (heritable) contributions to the expression decline. To characterise potential genetic factors underlying the low-fluorescence phenotypes observed after 140 generations, we again focused on the haploid High-2 and High-3 strains. As above, three cultures with the lowest production at the 140th generation were selected for each strain (High-2_culture 3, 5, and 8 and High-3_culture 2, 7 and 8). These cultures originated from the eight replicate cultures initially established for each strain in the long-term production experiment, but after 140 generations, they were no longer assumed to be clonal, because mutation and selection within each culture could have generated considerable genetic diversity.

**Fig. 6:**
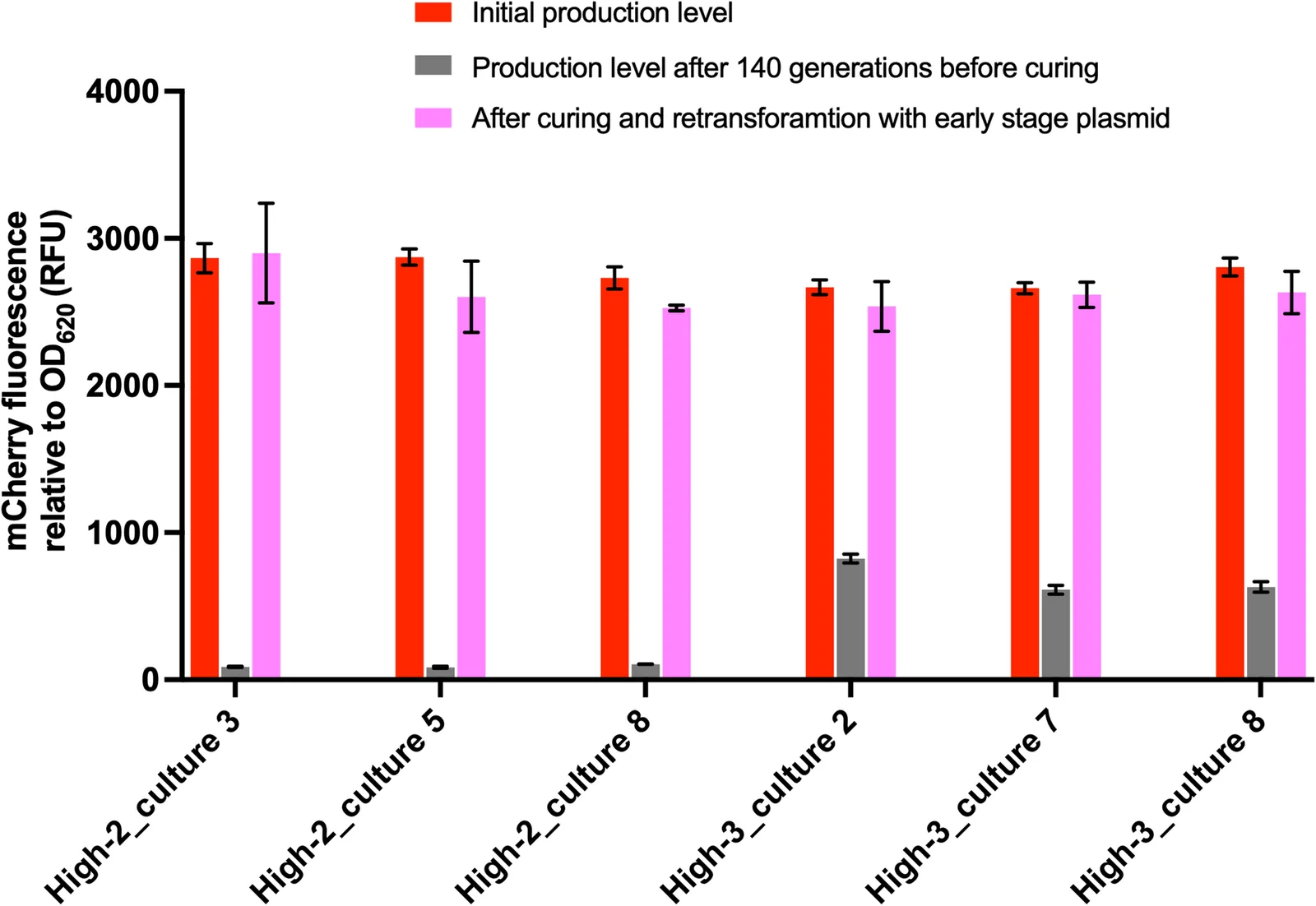
SARS-CoV-2-mCherry fusion protein expression by selected low-fluorescence colonies from High-2 and High-3 cultures after 140 generations, before and after curing and retransformation with early-stage plasmid. Initial fluorescence levels at the 10th (early stage, red bars) and 140th (late stage, grey bars) generations are shown as controls. The selected, cultured colonies from Figure S5 (red points) were cured of their production plasmid and retransformed with production plasmids extracted from the corresponding cultures at the 10th generation (initial high-production state). mCherry levels were measured after 3 days of culture and normalised to OD_620_. Pink bars represent fluorescence after curing and retransformation. Data are means ± SEM of three biological replicates for each selected colony

To select candidates for detailed analysis, 16 individual colonies derived from each of the above cultures were picked randomly, and their production after culturing was measured at single-colony resolution (Figure S5). From each set of 16, the colony with the lowest fluorescence (highlighted in red in Figure S5) was selected for further investigation. These colonies were cured of their plasmids by serial passaging in YPD medium and checking for lack of growth on media without supplement of leucine. In parallel, plasmids were extracted from High-2 and High-3 cells at an earlier stage of the long-term production experiment (10th generation), i.e., before fluorescence decline, and these plasmids were used to retransform the cured strains. Expression of the SARS-CoV-2-mCherry fusion by the cured strains after retransformation with these plasmids returned to the initial fluorescence level (Fig. 6). This suggested that, in the selected evolved low-fluorescence isolates analysed here, plasmid-associated changes were sufficient to explain the stable reduction in mCherry fluorescence. However, this does not exclude additional contributions from host physiology, epigenetic state or nuclear genomic changes in other cultures or at earlier stages of the process.

**Fig. 7:**
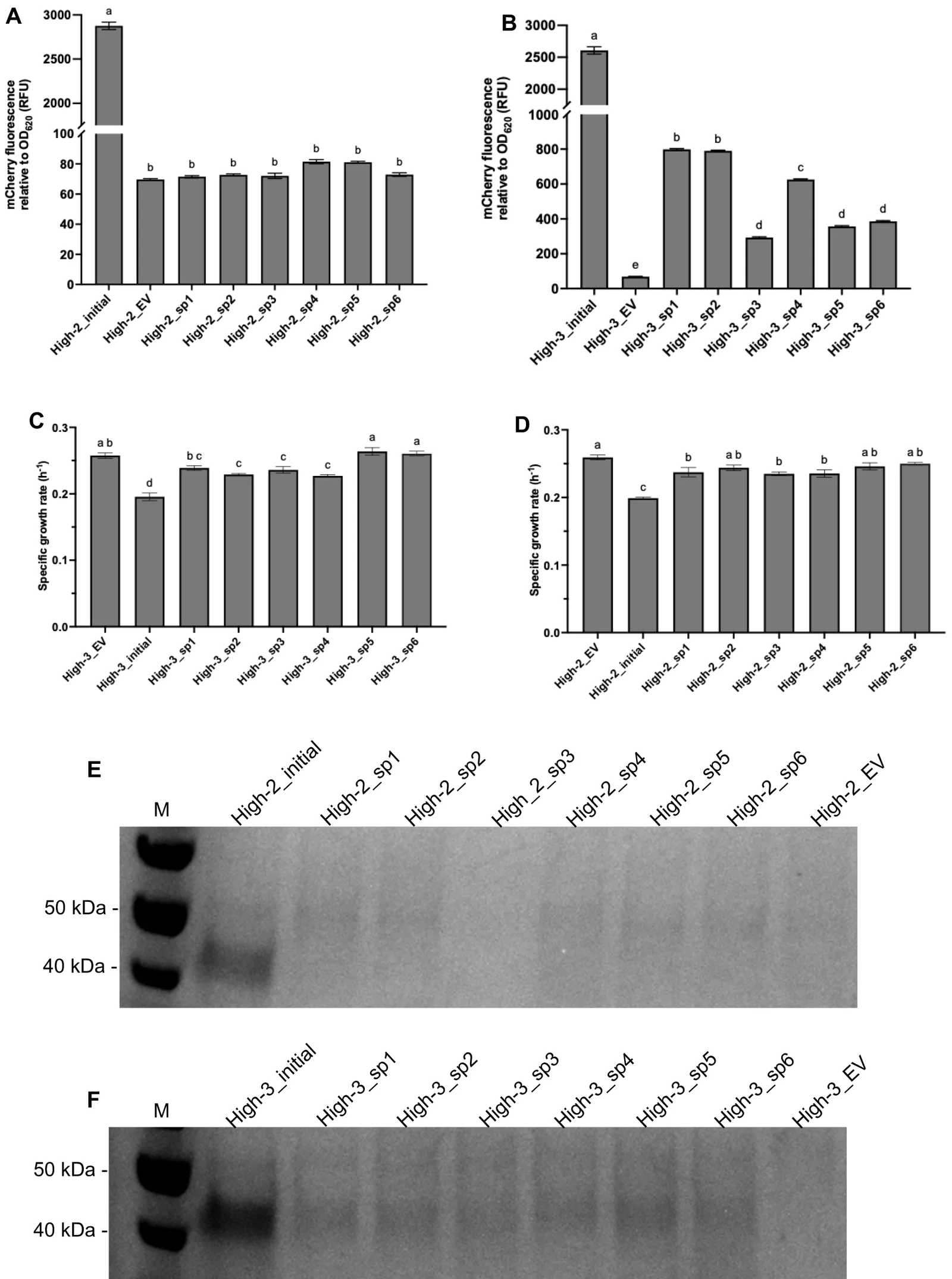
SARS-CoV-2-mCherry fusion protein expression and growth rates of retransformants carrying plasmids isolated from evolved low-fluorescence colonies. High-2 and High-3 strains at the early stage (10th generation) cured of plasmids were retransformed with plasmids extracted from selected colonies derived from evolved cultures at 140th generation (red data points in Figure S5). Two independent retransformants were selected from each plasmid extract and labelled as single-type plasmid retransformants, sp1 to sp6. The High-2 and High-3 cured strains retransformed with production plasmids extracted at the initial high-production, 10th generation stage (High-2_initial and High-3_initial) and empty vector (EV) lacking the production cassette (High-2_EV and High-3_EV) were used as controls. (A, B) SARS-CoV-2-mCherry fluorescence levels in High-2 (**A**) and High-3 (**B**) retransformants, measured as mCherry fluorescence normalised to OD_620_ after 3 days of culture in BMMD + CSM – Leu – Met medium. Data are presented as mean values ± SEM from three biological replicates. **C**, **D** specific growth rates corresponding High-2 (**C**) and High-3 (**D**) retransformants. Specific growth rates were calculated during the exponential growth phase using OD measurements taken over a 3-hour interval and presented as mean values ± SEM from four biological replicates. Means with different letters are significantly different (Ordinary one-way ANOVA, *p* < 0.05). **E**, **F** SDS-PAGE analysis of secreted SARS-CoV-2-mCherry fusion protein in culture supernatants from High-2 (**E**) and High-3 (**F**) retransformants grown for 3 days in BMMD + 2xCSM – Leu – Met medium. M, molecular weight marker

To characterise changes in the expression plasmid that might have contributed to the observed fluorescence decline, plasmids isolated from the selected low-fluorescence colonies were sequenced. Because individual plasmid molecules in a high-copy system can accumulate mutations independently during successive rounds of replication and segregation, we first extracted plasmids from the selected low-fluorescence colonies (yielding six plasmid preparations in total; three from High-2 and three from High-3 colonies) and retransformed these plasmids into the corresponding cured High-2 and High-3 strains derived from the 10th generation of the long-term production experiment. This provided an isogenic host background, allowing differences in mCherry fluorescence among retransformants to be attributed primarily to plasmid-associated differences. For each plasmid preparation, two independent retransformants were selected (resulting in six retransformants for High-2 and six for High-3), and each retransformant was presumed to carry a single-type plasmid (sp = single-type plasmid). The aim was not to achieve comprehensive coverage of all potential plasmid variants, but rather to indicate whether heterogeneity may exist within plasmid populations of individual colonies and to identify dominant changes associated with fluorescence decline. The retransformants were cultured in BMMD + CSM – Leu – Met medium for three days, and fluorescence levels were measured for each (Fig. 7A and B). Fluorescence levels from all the selected High-2 retransformants were comparable to the empty vector (i.e. non-fluorescent) control. In contrast, the High-3 retransformants displayed more variable fluorescence levels. The High-3_sp3 and sp4 retransformants had a significant difference in fluorescence despite both carrying plasmids originating from the same selected colony. This supported the suggestion that the plasmid populations within each selected colony might carry different mutations. Plasmid DNA was then re-extracted from these retransformants with the assumption that each retransformant only took up one plasmid from the extracted plasmid DNA. These were subjected to whole-plasmid sequencing to identify mutations that might explain the reduced-fluorescence phenotypes.

Whole-plasmid sequencing revealed that all the plasmids carried mutations, with some occurring in the same regions but varying in type across the plasmids (Table 1). Notably, even plasmids from the same colony carried different mutations, supporting the suggestion that multiple plasmid variants coexisted within individual cells at later stages of the bioproduction. All plasmids from evolved cultures of the High-2 strain (i.e. High-2_sp1-6) contained mutations within the expression cassette: five in the SARS-CoV-2 spike protein sequence and one in the mCherry sequence. Such mutations likely explain why all High-2 retransformants exhibited very low mCherry levels (Fig. 7A), as each should cause either a frameshift in the SARS-CoV-2 spike protein fragment sequence or premature termination of translation; either scenario is expected to decrease measurable mCherry fluorescence. For plasmids from the High-3 retransformants, the expression levels were much lower than the initial state, but three of them (High-3_sp1, 2, 4) conferred higher SARS-CoV-2-mCherry expression than the other three (High-3_sp3, 5, 6) (Fig. 7B). Notably, High-3_sp1 carried mutations only in the *FRT* sites, and High-3_sp3 harboured a mutation exclusively in the *MET17* promoter region, implying that the mutations at either of these locations can be sufficient to reduce production.

Given that recombinant protein production can elevate mutation rates under cellular stress [48–51], it cannot be completely ruled out that some mutations arose during later steps such as single-colony isolation, short-term culturing, or transformation. However, the reference plasmids from the 10th generation (High-2_initial and High-3_initial) were subjected to the same procedures of colony isolation, plasmid extraction, retransformation, re-extraction and sequencing. The mutations listed in Table 1 were identified by aligning the sequences of evolved plasmids from the 140th generation (High-2_sp1-6 and High-3_sp1-6 sequences) against these 10th -generation references. It is therefore very unlikely that the detected mutations resulted from artefacts introduced during those later steps, rather than during the prior long-term cultivation. The potential growth impact of production burden was also assessed by measuring the specific growth rates of strains at the initial (i.e. 10th generation) and late stages (after 140 generations) of the expression cycle and after retransformation (Fig. 7C and D). Strains characterised by high fluorescence at the initial stage exhibited an approximate 20% slower growth rate compared to the empty vector controls. However, the evolved low-fluorescence cultures at the 140th generation and the retransformants expressing low-fluorescence plasmids showed increased growth rate relative to the initial stage, which either matched or approached that of the empty vector controls. To examine whether the mutated variants might still express non-fluorescent proteins, we analysed culture supernatants from all High-2 and High-3 retransformants by SDS-PAGE. The results corroborated that protein expression was indeed reduced compared to the initial high production (Fig. 7E and F). These observations support the inference that declining fluorescence over time alleviated the metabolic burden on the cell.

**Table 1:** Nature of mutational changes characterised by sequencing of plasmids extracted from the indicated strains after expression for 140 generations (the specific sequence changes are detailed in Table S1)

Plasmids from strains after 140 generations of cultivation	Mutation types*	Locations of mutational changes	Predicted functional consequence
High-2_sp1 (from the selected colony of Culture 3)	type 1a, type 1c, type 2a, and type 8a	*FRT* regions, SARS-CoV-2 coding sequence, and noncoding region	Impaired *FLP*-mediated recombination; frameshift and premature disruption of the SARS-CoV-2-mCherry fusion protein
High-2_sp2 (from the selected colony of Culture 3)	type 2a	SARS-CoV-2 coding sequence	Frameshift and premature disruption of the SARS-CoV-2-mCherry fusion protein
High-2_sp3 (from the selected colony of Culture 5)	type 2b	SARS-CoV-2 coding sequence	Frameshift and premature disruption of the SARS-CoV-2-mCherry fusion protein
High-2_sp4 (from the selected colony of Culture 5)	type 1a, type 1c, type 2a, and type 8a	*FRT* regions, SARS-CoV-2 coding sequence, and noncoding region	Impaired *FLP*-mediated recombination; frameshift and premature disruption of the SARS-CoV-2-mCherry fusion protein
High-2_sp5 (from the selected colony of Culture 8)	type 2a	SARS-COV-2 coding sequence	Frameshift and premature disruption of the SARS-CoV-2-mCherry fusion protein
High-2_sp6 (from the selected colony of Culture 8)	type 4 and type 6	mCherry coding sequence and *FLP* coding sequence.	Frameshift and premature disruption of the SARS-CoV-2-mCherry fusion protein; impaired *FLP* function
High-3_sp1 (relatively high mCherry) (from the selected colony of Culture 2)	type 1e and type 1f	*FRT* regions	Impaired *FLP*-mediated recombination
High-3_sp2 (relatively high mCherry) (from the selected colony of Culture 2)	type 1b, type 5 and type 8c	*FRT* regions and terminator of expression cassette	Impaired *FLP*-mediated recombination; Impaired transcription termination or mRNA stability
High-3_sp3 (from the selected colony of Clone 7)	type 3	p*MET17* region	Impaired *pMET17* promoter activity
High-3_sp4 (relatively high mCherry) (from the selected colony of Clone 7)	type 1b, type 1d and type 8b	*FRT* regions and noncoding region	Impaired *FLP*-mediated recombination
High-3_sp5 (from the selected colony of Clone 8)	type3, type 7 and type 8a	p*MET17*, *LEU2* promoter, noncoding region	Impaired *pMET17* promoter activity; disrupted LEU2 expression or plasmid selection
High-3_sp6 (from the selected colony of Clone 8)	type 1a, type1c and type 3	*FRT* regions and p*MET17*	Impaired *FLP*-mediated recombination; Impaired *pMET17* promoter activity

*Mutation types refer to different regions of the plasmid where sequence changes were identified. Type 1: *FRT* regions; Type 2: SARS-CoV-2 spike protein fragment coding sequence; Type 3: *pMET17* promoter region; Type 4: *mCherry* coding sequence; Type 5: terminator region of the expression cassette (downstream of mCherry); Type 6: *FLP* recombinase coding sequence; Type 7: *LEU2* promoter region; Type 8: Non-coding regions. The lower-case letters a, b, c, d, e and f indicate distinct mutations within that region. Detailed sequence changes corresponding to each mutation type and subtype are provided in Table S1

### Mutations in the *MET17* promoter and *FRT* sites impair promoter activity or alter plasmid copy number

**Fig. 8:**
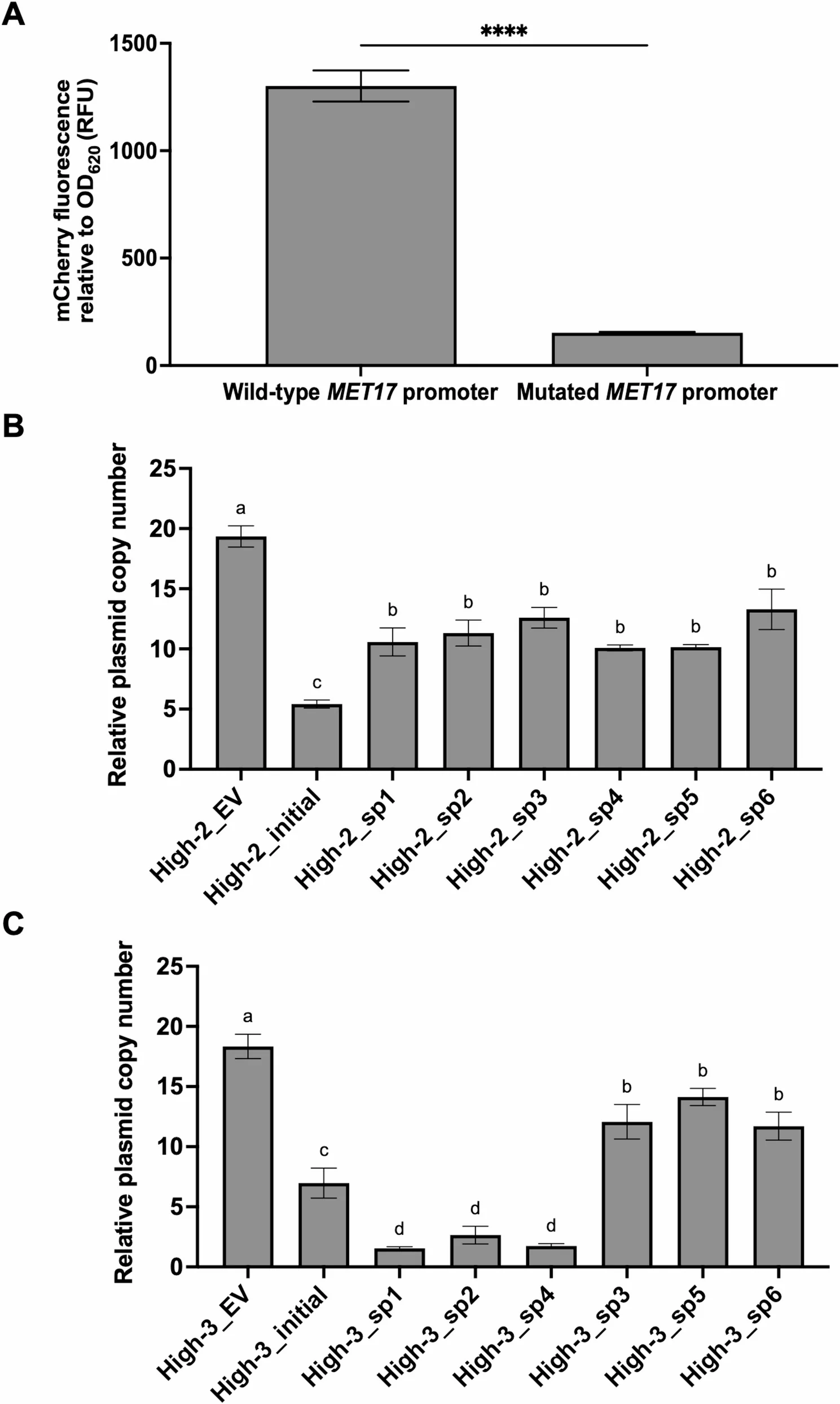
Impact of *MET17* promoter and *FRT-*site mutations on expression and plasmid copy number. Data represent mean values ± SEM of three biological replicates. **A** Comparison of wild-type and mutated *MET17* promoter activity. Cells were grown overnight in YNB broth lacking leucine. The mutated *MET17* promoter contains a single G-A point mutation at the CACGT**G** promoter motif, changing it to CACGT**A**. Reporter plasmids expressing mCherry under the indicated promoters were transformed into *S. cerevisiae* BY4741. Promoter activity was quantified by mCherry fluorescence normalised to OD_620_. Statistical significance (unpaired *t-*test): *p* < 0.0001 (****). **B**, **C** Plasmid copy number per genome in High-2 (**B**) and High-3 (**C**) retransformants carrying mutated plasmids (High-2_sp1-6, High-3_sp1-6) determined by ddPCR. Empty vector (EV) and initial high-production plasmid from the 10th generation were used as controls. The *ACT1* gene was used as a genomic reference. Relative copy numbers were calculated as the ratio of plasmid concentration (copies/µL) relative to genomic DNA concentration (copies/µL). Means with different letters are significantly different (Ordinary one-way ANOVA, *p* < 0.05)

To substantiate the genetic basis of the fluorescence decline observed in evolved plasmids, we examined the mutations identified in the regulatory *MET17* promoter and the *FRT* sites of the production plasmids. Whole-plasmid sequencing revealed that High-3_sp3 only contained a single nucleotide G-to-A point mutation within the CACGT**G** motif in the *MET17* promoter, and the same mutation was also identified in the *MET17* promoter of High-3_sp5 and sp6. (Table 1 and Table S1). This motif serves as the binding site for Cbf1, a transcription factor that supports transcriptional activation of *MET17* under methionine limitation [52]. Strains carrying these plasmids exhibited markedly reduced mCherry expression compared to those carrying evolved plasmids with an unmutated *MET17* promoter (i.e. High-3_sp1, sp2 or sp4). It was therefore hypothesised that this substitution impaired promoter activity. To test this, the wild-type and mutated *MET17* promoter sequences were cloned upstream of an mCherry reporter in the centromeric plasmid pRS315. Reporter assays showed that the G-to-A mutation reduced *MET17* promoter activity by approximately 88% (Fig. 8A).

We next investigated whether the mutations identified in the *FRT* sites affected plasmid copy number. The 2-micron plasmid in yeast depends on a site-specific recombination system involving the FLP recombinase and its target sites (*FRT*s) to maintain high plasmid copy number [53]. Therefore, sequence changes in these regions could potentially disrupt plasmid amplification. To test this, plasmid copy number was quantified in the High-2 and High-3 retransformants using droplet digital PCR (ddPCR). In the High-2 background, retransformants carrying evolved plasmids (High-2_sp1-6) showed higher copy numbers than the initial production plasmid but remained below the empty-vector control (Fig. 8B). In contrast, the High-3 retransformants displayed a clearer link between copy number and fluorescence: plasmids only containing functional mutations in the *FRT* sites (i.e. High-3_sp1, sp2 and sp4) showed lower copy numbers but higher fluorescence, whereas those with additional *MET*17 promoter mutation (i.e. High-3_sp3, sp5 and sp6) had both reduced fluorescence and elevated copy number (Figs. 7B and Fig. 8C). These results suggested that the mutations in the *FRT* sites can be associated with reduced plasmid copy number, but that copy number is not determined by *FRT* sequence alone. Instead, plasmid copy number may also be influenced by expression burden or other plasmid-associated changes. One possible explanation is that reduced transcription from the recombinant expression cassette, for example, due to the *MET17* promoter mutation, may alleviate interference with native 2-micron plasmid biology or reduced expression-associated burden, thereby permitting higher copy number. However, this remains a speculative model and would require further experimental testing.

**Fig. 9:**
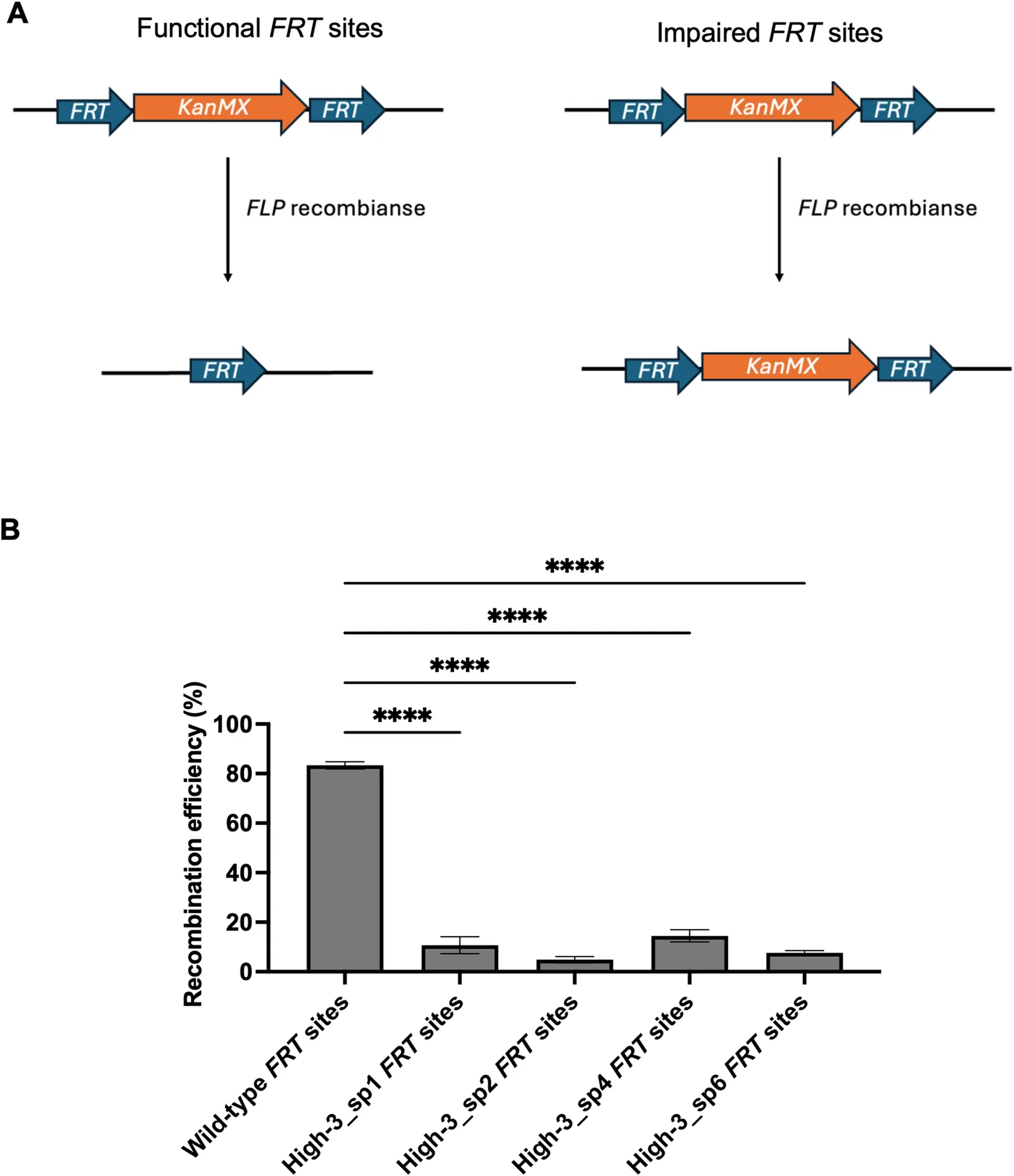
Recombination efficiency of *FLP* recombinase with original and mutated *FRT* sites. **A** Design principle for testing *FLP*-mediated recombination efficiency. Plasmids were constructed with a *KanMX* resistance gene flanked by either functional (left) or mutated (right) *FRT* sites arranged in the same direction. Upon expression of *FLP* recombinase, recombination between functional *FRT* sites excises the *KanMX* cassette, leading to loss of G418 resistance; plasmids with impaired *FRT* sites do not support *FLP*-mediated recombination, and the *KanMX* gene is retained, maintaining G418 resistance. **B** The efficiency of *FLP*-mediated recombination with the original and the mutated *FRT* sites derived from High-3_sp1, sp2, sp3 and sp6 plasmids. The recombination efficiency was assessed by comparing colony counts on YPD and YPD+G418 plates. Data represent mean values ± SEM of three biological replicates. Statistical significance: *p* < 0.0001 (****)

To substantiate the effects of the *FRT* mutations on plasmid copy number, we constructed plasmids containing a kanamycin (G418) resistance gene (*KanMX*) flanked by mutated minimal *FRT* sequences arranged in the same direction (Fig. 9A). All four types of mutated *FRT* sites derived from High-3_sp1, sp2, sp4 and sp6 plasmids were tested (Table 1 and Table S1). These constructed plasmids were transformed into a strain of *S. cerevisiae* BY4741 that had been engineered to overexpress FLP recombinase under the control of the galactose-inducible strong promoter *pGAL1* (see Methods for details). Upon induction, active FLP should catalyse recombination between functional *FRT* sites, resulting in excision of the *KanMX* cassette and G418 sensitivity. However, where *FRT* mutations disrupt recombination, the cassette remains intact, and the transformants retain G418 resistance, indicating impaired *FRT* activity (Fig. 9A). As expected, the control construct containing the wild-type *FRT* sites exhibited high recombination efficiency (Fig. 9B), i.e., successful excision of the *KanMX* cassette. In contrast, all four mutated *FRT* variants showed significantly reduced recombination efficiencies, ranging from 3% to 21.5% of the control, indicating that these mutations impaired FLP recognition or recombination activity.

## Discussion

Sustained recombinant protein production in yeast cell factories is highly desirable for industrial biomanufacturing, yet maintaining productivity over extended production campaigns remains a major challenge [54]. In this study, we observed that different recombinant proteins impose varying burdens on the host: intracellular AP205 VLP production remained stable for at least 140 generations, whereas high-level expression of a secreted SARS-CoV-2 spike protein fragment tended to decline rapidly. This contrast indicates that protein-specific properties, such as folding demands, cellular localisation, potential toxicity, etc., may play critical roles in production stability and strain performance. Using the secreted SARS-CoV-2-mCherry fusion protein as a model, we investigated how high-level recombinant protein production is influenced by non-genetic heterogeneity and shapes plasmid evolution within the overall dynamics of expression decline.

While high-fluorescence strains generally exhibited a rapid decline in productivity over time, and our study focused on such cases, it is notable that certain strains screened from the libraries (e.g. High-1) maintained moderate-to-high fluorescence levels over the 140-generation cultivation period (Figs. 2A and Fig. 3). This highlights the underlying diversity in the starting strain pool, where rare genotypes possess both high fluorescence capacity and resilience to production-associated stresses. These ‘stay-high’ strains demonstrate that it is indeed possible to achieve durable productivity under prolonged biomanufacturing conditions, and their existence underscores the value of large-scale library screening of genetically diverse populations for isolating robust production strains.

Three fluorescence-defined subpopulations, non-fluorescent, low-fluorescence and high-fluorescence were observed during the early production cycle in both High-2 and High-3 strains via FACS. Because the SARS-CoV-2-mCherry fusion protein is secreted, these subpopulations should be interpreted as distinct intracellular mCherry fluorescence states rather than direct measures of total protein production. Phenotypic heterogeneity such as this is prevalent for almost any phenotype within genetically uniform cell populations and can represent a bet-hedging strategy against potential future perturbations, such as from environmental stress [14, 55–58]. Our study extends the relevance of this concept to recombinant protein expression systems, suggesting that the existence of diverse expression phenotypes may benefit the organism via persistence of lower-fluorescence cells with lower expression-related burdens. This phenotypic heterogeneity could reflect stochastic gene expression or more deterministic regulatory factors like cell cycle-stage heterogeneity, consistent with previous observations of bet-hedging in microbial systems [59, 60]. In our system, such non-genetic diversity may contribute to the early enrichment of low-fluorescence cells that experience less burden and grow faster, thereby potentially shaping the subsequent trajectory of genetic adaptation.

Whole plasmid sequencing of plasmids from selected colonies derived from low-fluorescence cultures at the 140th generation revealed frequent mutations in the SARS-CoV-2 spike protein fragment coding sequence, the *MET17* promoter region and the *FRT* sites of the 2-micron plasmid backbone (Table 1). Strikingly, three out of eight cultures from both High-2 and High-3 strains exhibited a pronounced decline in productivity, despite originating from the same starting colony. This divergence in production is consistent with mutations occurring stochastically during prolonged cultivation. Sequencing of evolved plasmids from representative low-fluorescence colonies derived from those low-fluorescence cultures revealed non-identical mutational profiles across samples. Although sampling was limited (one low-fluorescence, culture-derived colony selected from each of three evolved cultures per strain, and two plasmids sequenced per colony; six plasmids in total for each strain, High-2_sp1-6 and High-3_sp1-6), each plasmid displayed a distinct mutation pattern (Table 1 and Table S1). This observation supports the idea that individual cells within the same population can accumulate distinct mutations over time, resulting in intra-population heterogeneity in plasmid genotypes. Similar patterns of intra-populational mutations have been observed in previous studies, where functionally convergent outcomes arise through diverse mutational trajectories [61, 62]. Moreover, our data also showed that even within a single colony derived from the late production cycle, plasmids may carry heterogeneous mutations (Table 1 and Table S1), implying that plasmid populations within single cells can diverge over generations. This highlights the potential for intracellular plasmid heterogeneity to emerge during prolonged cultivation, which is an underappreciated source of evolutionary diversification in multi-copy plasmid-based expression systems. It is plausible that a broader spectrum of mutational heterogeneity exists within each culture; further sequencing of additional colonies and plasmids would likely uncover even more diversity in plasmid evolution.

A limitation of the present study is that the temporal sequence linking early non-genetic fluorescence heterogeneity, growth advantage and plasmid mutation was not directly reconstructed. Although the data support a model in which lower-fluorescence states are associated with faster growth and in which evolved low-fluorescence isolates carry plasmid variants that reduce expression, we did not perform population-level plasmid sequencing across intermediate generations. Therefore, the data do not show when specific plasmid variants first arose, how their frequencies changed over time, or whether their expansion occurred before or after enrichment of lower-fluorescence cellular states. In addition, plasmid sequencing was performed on selected low-fluorescence endpoint isolates, which was useful for identifying plasmid-associated mechanisms of stable fluorescence loss but naturally enriched the analysis for severe loss-off-expression genotypes. As a result, these data do not allow estimation of mutation frequencies across the whole evolved population. Future work using population-level plasmid sequencing at multiple time points, ideally including stable high-fluorescence lineages (such as the High-1 strain here) and combined with single-cell fluorescence analysis, would provide a stronger comparison between stable and unstable expression outcomes and help resolve the timing and frequency of plasmid variant emergence during prolonged recombinant protein expression.

It is noted that the long-term sequential passaging system used here should be interpreted as a laboratory model for production-associated selection rather than a direct simulation of industrial continuous fermentation. The 48-well plate format and repeated batch culture with stationary-phase passaging confer selective pressures that differ from those experienced in controlled bioreactor operation. For example, nutrient depletion, changing growth phase, oxygen transfer and repeated dilution could affect the enrichment of lower-fluorescence or faster-growing subpopulations. Nevertheless, this system provides a useful and experimentally tractable approach for comparing long-term fluorescence-based production stability across genetically diverse yeast strains and for identifying phenotypic or plasmid-associated changes that arise under sustained recombinant protein expression.

In yeast-based systems, regulatory promoters are widely employed for the rational design and optimisation of cell factories [63]. In the present system, expression of the SARS-CoV-2-mCherry fusion protein was driven by the methionine-regulated *MET17* promoter. Our findings demonstrated that the CACGTG motif recognised by yeast centromere binding factor I (Cbf1) is essential for the full activity of the *MET17* promoter. This is consistent with previous work showing that Cbf1 is essential for the full induction of *MET17* transcription in response to methionine limitation [64]. In addition to mutation of the promoter, we identified the 8-bp spacer region of the *FRT* sites as a mutational hotspot in the present expression system. This region is central to strand pairing, crossover and cleavage in the FLP-mediated amplification in the whole-2-micron plasmid-based system [65]. Although earlier studies suggested that certain substitutions or even single-nucleotide insertions or deletions in the spacer region can still support FLP-mediated recombination [66–68], our results indicated that the recombination is impaired when this spacer sequence is excessively altered. Specifically, all tested *FRT* mutants (i.e. High-3_sp1,2,4,6 plasmids) harboured non-identical *FRT* sites, with at least one spacer length exceeding 10 bp, which likely compromised the FLP-mediated recombination efficiency. Previous work has shown that functional FLP-mediated recombination is generally limited to *FRT* sites with spacer lengths of less than 10 bp [66]. However, the relationship between *FRT* mutation, plasmid copy number and mCherry fluorescence was not linear. Some evolved plasmids with mutations in the *FRT* sites showed reduced copy number, consistent with impaired *FLP-*mediated amplification, whereas other low-fluorescence plasmids, particularly those carrying the *MET17* promoter mutation, showed elevated copy number. Therefore, we interpret *FRT* mutations as one mechanism that can impair recombination and contribute to altered plasmid behaviour, while recognising that plasmid copy number and recombinant protein expression are likely shaped by multiple factors, including *FRT* functionality, expression-associated burden and other plasmid-associated changes.

In conclusion, this case study provides a mechanistic view of one route by which fluorescence-based productivity can decline during extended heterologous protein expression in yeast. Early in the production campaign, cultures expressing the secreted SARS-CoV-2-mCherry fusion protein contained substantial non-genetic heterogeneity in intracellular mCherry fluorescence, with only a minority of cells showing high intracellular fluorescence at any time. Cells or cultures with lower fluorescence showed faster growth, suggesting that reduced expression or reduced intracellular burden may provide a selective advantage during prolonged cultivation. At the endpoint of prolonged cultivation, evolved low-fluorescence isolates carried diverse plasmid mutations, including changes predicted to reduce expression and alleviate burden. Together, these findings support a model in which reduced expression can provide a selective advantage during prolonged cultivation, and in which plasmid-associated changes can stabilise low-expression states in evolved isolates. From an applied perspective, our findings indicate that improved long-term stability for burdensome recombinant protein expression in *S. cerevisiae* may be achieved by appropriate selection of strains from genetically diverse populations.

## Electronic Supplementary Material

Below is the link to the electronic supplementary material.

Supplementary Material 1.

## Data Availability

All data generated or analysed in this study are included in the article, supplementary files, or available on request.
