# Supplementary material for: The impact of evolutionary dynamics and expression plasmid stability on long-term heterologous protein production by yeast
Source: Microb Cell Fact. 2026 Jul 23;25:218. doi: 10.1186/s12934-026-03071-1 (PMC13682124; doi:10.1186/s12934-026-03071-1)
Supplement: Supplementary file 1 [file 12934_2026_3071_MOESM1_ESM.docx]

**Supplementary Materials**

**A**

**B**

**Figure S1: mCherry-tagged product expression in selected S. cerevisiae strains from haploid and diploid libraries.** (A) Expression of 36 haploid and 36 diploid strains expressing the AP205-VLP-mCherry fusion protein. (B) Expression of 36 haploid and 36 diploid strains expressing SARS-CoV-2-mCherry fusion protein. mCherry fluorescence was measured using a Tecan plate reader (different from the plate reader used for the main results). Each data point represents an individual strain.

**A**

**B**

c

c

a

a

b

b

b

b

b

b

**Figure S2: Heterogeneity level of mCherry fluorescence in High-2 and High-3 initial cultures or the evolved low-fluorescence cultures, measured by coefficient of variation.** Cell-to-cell heterogeneity level in SARS-CoV-2-mCherry fusion protein expression was assessed using the coefficient of variation (CV) of single-cell mCherry fluorescence for High-2 (A) and High-3 (B) cultures at the 10^th^ generation (High-2_initial and High-3_initial) or the low-fluorescence cultures at the 140^th^ generation (High-2_Culture 3, 5 and 8, and High-3_Culture 2, 7 and 8). Data were obtained by flow cytometry. Bars represent means ± SEM of three biological replicates, each biological replicate based on measurements from 10,000 cells. Means with different letters are significantly different (Ordinary one-way ANOVA, p < 0.05).

**A**

**B**

**Figure S3: SARS-CoV-2-mCherry expression levels following regrowth of sorted subpopulations.**

Fluorescence levels of the SARS-CoV-2-mCherry fusion protein were measured in sorted subpopulations of High-2 (A) and High-3 (B) carrying the initial production plasmid. The original unsorted cultures (High-2_initial and High-3_initial) were used as controls. Approximately 15,000 cells were sorted from each of the non-fluorescent, low-fluorescence and high-fluorescence subpopulations based on mCherry fluorescence and cultured in BMMD + CSM – Leu – Met medium for three days. Fluorescence intensity was then quantified using a plate reader and normalised to OD_620_. Data represent mean values ± SEM of six biological replicates.

**Non-fluorescent**

**Low-fluorescence**

**High-fluorescence**

Cell count

**A**

Cell count

Cell count

**B**

High-2_Non-fluorescent

High-2_Low-fluorescence

High-2_High-fluorescence

High-3_Non-fluorescent

High-3_Low-fluorescence

High-3_High-fluorescence

Sorted_High-fluorescence

Sorted_Low-fluorescence

Sorted_non-fluorescent

**Figure S4: SARS-CoV-2-mCherry fusion protein expression at the single-cell level following cell sorting and regrowth of subpopulations.**

Approximately 15,000 cells were isolated from each subpopulation (non-fluorescent, low-fluorescence, and high-fluorescence) of High-2 and High-3 strains carrying the initial production plasmid based on mCherry fluorescence normalised to cell size (FSC-A). (A) Sorted cells were regrown in BMMD + CSM – Leu – Met medium, and their SARS-CoV-2-mCherry fusion protein fluorescence were re-analysed by flow cytometry (CytoFLEX S Flow Cytometer). Each distribution represents data from 10,000 individual cells. (B) Post-sort reanalysis performed immediately after sorting (Astrios Cell Sorter) confirmed that the sorted populations were highly enriched within their respective fluorescence ranges defined by gating. Note: data in panels A and B were obtained using different flow cytometers, and mCherry values are therefore not directly comparable.

**Figure S5: Expression of the SARS-CoV-2-mCherry fusion protein from individual colonies isolated from low-fluorescence evolved cultures after 140 generations of cultivation.** Sixteen individual colonies from each evolved culture were grown in 48-well microtiter plates in 500 µL BMMD + CSM – Leu – Met medium for 3 days, before measuring cellular mCherry fluorescence with normalisation to OD_620_. Shown are the SARS-CoV-2-mCherry fusion protein production levels of each of the 16 colonies isolated from each of the three lowest-expressing High-2 evolved cultures (culture 3, 5 and 8) and the three lowest-expressing High-3 evolved cultures (culture 2, 7 and 8) after 140 generations. Each data point is the mean of approximately 1.5 x 10^8^ cells analysed. Red data points indicate colonies selected for subsequent retransformation and plasmid analysis tests.

**Table S1: Specific sequence changes identified by whole plasmid sequencing (see Table 1 for summary of the changes)**

| **Types of mutation (description)** |  | **10^th^ generation plasmid sequence** | **140^th^ generation plasmid sequence** |
| --- | --- | --- | --- |
| Type 1 (at *FRT* sites) | Type 1a (at *FRT* site upstream of the FLP; four nt addition) spacer from 8bp to 12 bp | TCTAG----AAA | TCTAGCTAGAAA |
|  | Type 1b (at *FRT* site upstream of the FLP; two nt addition) spacer from 8bp to 10 bp | TCT--AGAAA | TCTCTAGAAA |
|  | Type 1c (at *FRT* site downstream of the FLP; four nt deletion) spacer from 8bp to 4 bp | TTTCTAGA | TTT----A |
|  | Type 1d (at *FRT* site downstream of the FLP; two nt addition) spacer from 8bp to 10 bp | TTTCTAGA--GAA | TTTCTAGAGAGAA |
|  | Type 1e (at *FRT* site upstream of the FLP; five nt addition) spacer from 8bp to 13bp spacer | TCT-----AGAAA | TCTCTAGCTAGAAA |
|  | Type 1f (at *FRT* site downstream of the FLP; two nt deletion) spacer from 8bp to 6bp | TTTCTAGA | TTT--AGA |
| Type 2 (at SARS-COV-2 spike protein fragment sequence) | Type 2a (four substitution, one addition) | CAATCCTACGGCTTTC-AACC | CAATCCTACGTCCATGTAACC |
|  | Type 2b (four deletion, five addition) | CAATCCTACGGCTTTC-----AACC | CAATCCTACG----TCCATGTAACC |
| Type 3 (G to A SNP at the *pMET17* sequence) |  | CACGTGAAGCTGTCGATA | CACGTAAAGCTGTCGATA |
| Type 4 (at the mCherry sequence; nonsense mutation) |  | ATGGTCTCCAAAGGTGAAG | ATGGTCTCCAAAGGTTAAG |
| Type 5 (at terminator region of the expression cassette; downstream of mCherry sequence; large deletion) |  | ACACCTCTACCGGCATGCCGAGCAAATGCCTGCAAATCGCTCCCCATTTCACCCAATTGTAGATATGCTAACTCCAGCAATGAGTTGATGAATCTCGGTGTGTATTTTATGTCCTCAGAGGACAACACCTGTTGTAA | ACAC----ACCG-CATCAC----AAATGCCT-CAAATCGCT--------------------------------------------------------------------------------------------GTAA |
| Type 6 (at FLP region; a part of FLP sequence duplicated) |  | TCCCAGATACTTATTTTGGACTAATTT- | TCCCAGATACTTATTTTGGACTAATTTAAATGATTTCGGATCAACGTTCTTAATATCGCTGAATCTTCCACAATTGATGAAAGTAGCTAGGAAGAGGAATTGGTATAAAGTTTTTGTTTTTGTAAATCTCGAAGTATACTCAAACGAATTTAGTATTTTCTCAGTGATCTCCCAGATGCTTTCACCCTCACTTAGAAGTGCTTTAAGCATTTTTTTACTGTGGCTATTTCCCTTATCTGCTTCTTCCGATGATTCGAACTGTAATTGCAAACTACTTACAATATCAGTGATATTTTGGACTAATTT |
| Type 7 (insertion at *LEU2* promoter region) |  | GAGAACA-----------CACAGGGGCGCTATCGCAC | GAGAACATAACGAGAACACACAGGGGCGCTATCGCAC |
| Type 8 (at noncoding regions) | Type 8a (at the noncoding region upstream of the expression cassette; one nt deletion) | TAAAAAAAAAAAAATAC | TAAAAAAAAAAAA-TAC |
|  | Type 8b (at the noncoding region upstream of the expression cassette; one nt insertion) | TAAAAAAAAAAAAA-TAC | TAAAAAAAAAAAAAATAC |
|  | Type 8c (at the noncoding region downstream of the expression cassette; one nt deletion) | TTTTCGTATTG | TTTT-GTATTG |
